# Recent advances in liver‐on‐chips: Design, fabrication, and applications

**DOI:** 10.1002/SMMD.20220010

**Published:** 2023-02-12

**Authors:** Linjie Qiu, Bin Kong, Tiantian Kong, Huan Wang

**Affiliations:** ^1^ The Eighth Affiliated Hospital Sun Yat‐Sen University Shenzhen China; ^2^ School of Medicine Sun Yat‐Sen University Shenzhen China; ^3^ Guangdong Key Laboratory for Biomedical Measurements and Ultrasound Imaging Department of Biomedical Engineering School of Medicine Shenzhen University Shenzhen China

**Keywords:** biomimicry, cell culture, liver‐on‐chips, liver model, microfabrication

## Abstract

The liver is a multifunctional organ and the metabolic center of the human body. Most drugs and toxins are metabolized in the liver, resulting in varying degrees of hepatotoxicity. The damage of liver will seriously affect human health, so it is very important to study the prevention and treatment of liver diseases. At present, there are many research studies in this field. However, most of them are based on animal models, which are limited by the time‐consuming processes and species difference between human and animals. In recent years, liver‐on‐chips have emerged and developed rapidly and are expected to replace animal models. Liver‐on‐chips refer to the use of a small number of liver cells on the chips to simulate the liver microenvironment and ultrastructure in vivo. They hold extensive applications in multiple fields by reproducing the unique physiological functions of the liver in vitro. In this review, we first introduced the physiology and pathology of liver and then described the cell system of liver‐on‐chips, the chip‐based liver models, and the applications of liver‐on‐chips in liver transplantation, drug screening, and metabolic evaluation. Finally, we discussed the currently encountered challenges and future trends in liver‐on‐chips.

## Key points

- Liver physiology and pathology were reviewed.
- The liver‐on‐chips culture environment and cell system classification were summarized.
- The liver‐on‐chips applications in multiple scenarios were discussed.
- The challenges and outlook of liver‐on‐chips were provided.

## Graphical abstract

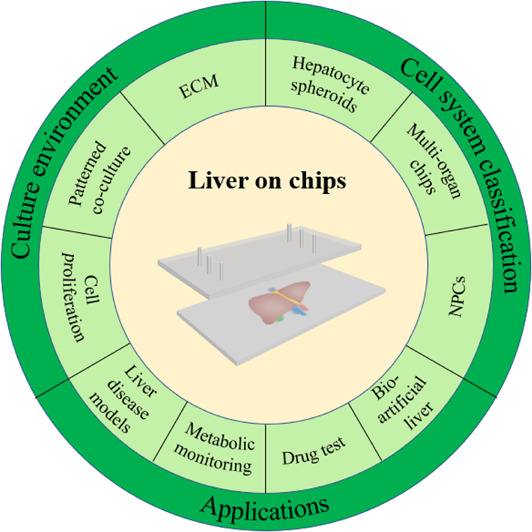

Liver‐on‐chips refer to the use of a small number of liver cells on the chips to simulate the liver microenvironment and ultrastructure in vivo. There are various kinds of liver‐on‐chips that have been developed, and these liver‐on‐chips can be divided into different types according to the culture environment, cell system, and intro model. These liver‐on‐chips have been explored for liver disease models, metabolic monitoring drug test, and bioartificial liver.

## 1 INTRODUCTION

The liver is the largest internal organ of the human body, accounting for approximately 2% of the body weight in adults; it plays an important role in maintaining normal life activities of the human body and has endocrine, exocrine, detoxification, hormone synthesis, and other functions.^1^, ^2^, ^3^ Although under normal circumstances, the liver has a powerful regenerative capacity in response to physical or chemical insults, in fact, over 900 drugs^4^ and toxins have been reported to cause varying degrees of damage to the liver, which impair the liver's ability to perform its normal physiological functions.^5^, ^6^, ^7^ Currently, animal models have been widely used to study liver physiological functions and drug toxicity experiments, and are recognized methods for evaluating drug liver injury.^8^, ^9^, ^10^, ^11^, ^12^, ^13^, ^14^, ^15^ However, genetic and metabolic differences between animals and humans often lead to unsatisfactory final results. About half of the drugs that do not cause liver damage in animal models are reported to cause liver damage in clinical practice.^16^, ^17^ Traditional 2D cultures can serve as an alternative to animal models to some extent but suffer from a lack of cell–cell interaction and a too‐rapid loss of cellular activity.^18^, ^19^ Therefore, it has become an increasingly popular topic to establish more accurate and bionic in vitro liver models.^20^, ^21^, ^22^

In recent years, to overcome the above‐mentioned shortcomings, people have explored liver‐on‐chips,^23^, ^24^, ^25^ which are novel liver cell culture platforms that mimic the key structure and core functions of the liver. The liver‐on‐chips consist of real living human hepatocytes, and those commonly used include primary human cells, immortalized human cell lines, and others. Hepatocytes are seeded in engineered systems that reliably recapitulate liver structure and functions. Most liver‐on‐chips have three things in common: 3D arrangement, multicellular species, and mechanical forces associated with the chip model. With the help of microfabrication technologies, a variety of liver‐on‐chips with different microstructures have been developed for mimicking the 3D hepatocyte culture environments.^26^, ^27^ By controlling the type and proportion of cells cultured, the chips can more accurately mimic the real liver. Additionally, microfluidic technologies, which use the microfluidic devices with microchannels and can regulate fluids in microscale precisely,^28^, ^29^, ^30^, ^31^, ^32^, ^33^, ^34^, ^35^, ^36^, ^37^, ^38^, ^39^, ^40^, ^41^, ^42^, ^43^, ^44^, ^45^, ^46^, ^47^, ^48^, ^49^, ^50^, ^51^, ^52^, ^53^, ^54^, ^55^, ^56^, ^57^, ^58^, ^59^ are often employed to construct the chips,^60^, ^61^, ^62^, ^63^, ^64^, ^65^, ^66^ and they can not only supply nutrients and excrete metabolites but also provide shear force for the cultured cells. Relative to traditional culture methods, hepatocytes can maintain phenotypes for longer periods of time, enabling previously elusive functions such as microstructural mimicry of liver sinusoids, hepatic lobules, and integration of multiple organ functions. More importantly, the liver‐on‐chips showed great potential in drug toxicity testing, promising to replace the use of animal models.^5^, ^67^ Therefore, researchers are constantly innovating the liver‐on‐chips to construct different types and functions of liver models.^68^, ^69^, ^70^

**FIGURE 1:**
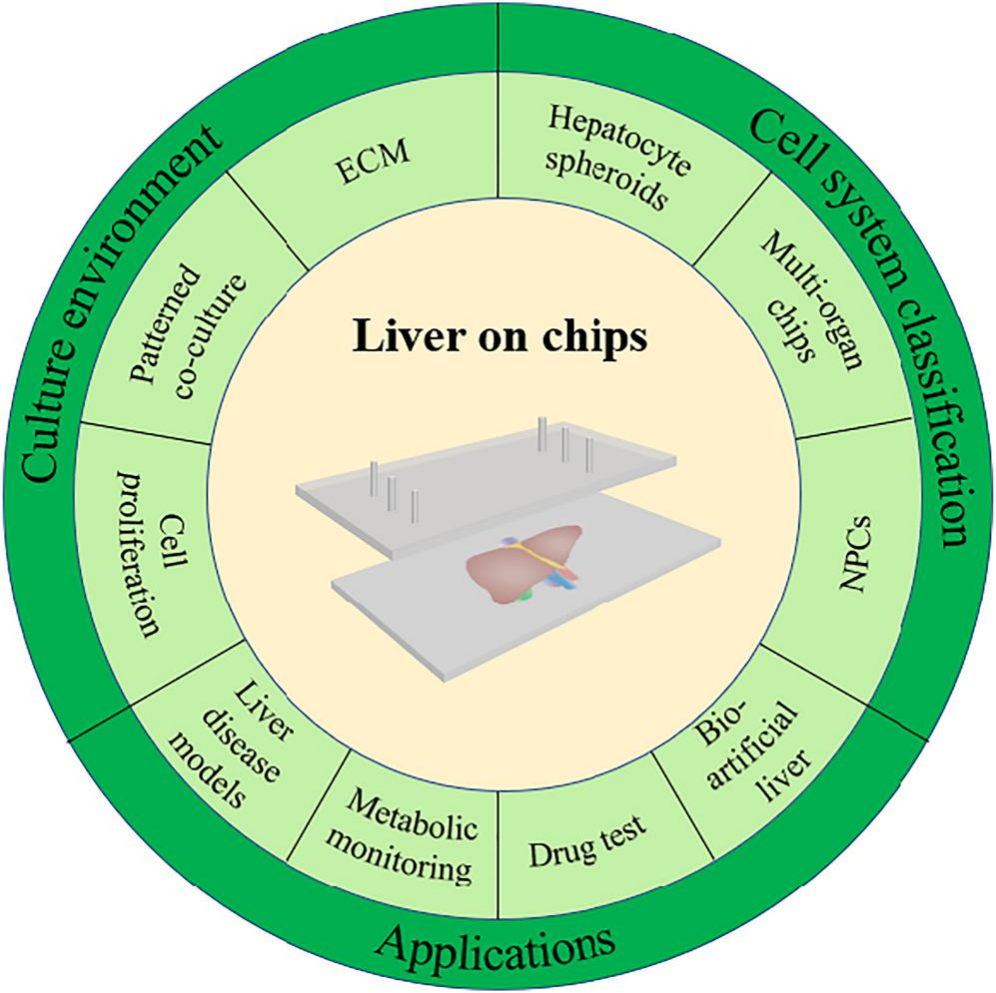
This TOC describes the main content of this review. We first introduce several aspects of the physiology and pathology of the liver, and then, we focus on the classification of liver‐on‐chips models through representative examples, ranging from culture environment to cell system classification. We elaborate on examples of applications of liver‐on‐chips in multiple fields. Finally, we summarize the remaining challenges and give an outlook on the future of the liver‐on‐chips.

In this review, we outline recent advances in liver‐on‐chips research (Figure 1). We discuss the variety of liver‐on‐chips, cell culture systems, and describe the advantages and disadvantages of each model. Next, we introduce the simulation objects of the liver‐on‐chips, including normal liver, liver cancer, and nonalcoholic fatty liver disease (NAFLD). Finally, we also summarize the challenges facing the current research, propose possible solutions, and provide an outlook on the future of liver liver‐on‐chips.

## 2 LIVER BIOLOGY AND PATHOLOGY

### 2.1 Biology

The liver is the largest parenchymatous organ of the human body. The whole liver presents an irregular wedge shape and is divided into a right lobe and a left lobe by the falciform ligament in the anterior wall of the liver.^71^, ^72^ Couinaud divides the liver into eight segments clockwise according to the branches of portal vein (PV), and each segment has its own inflow and outflow vessels and biliary system.^73^, ^74^ The liver is supplied by the PV, which carries blood rich in nutrients and toxins from the stomach, intestines, and spleen, and the hepatic artery, which provides oxygen‐rich blood.^75^ The surface of the liver is covered with a thin membrane of dense connective tissue. The membrane penetrates the liver to form a reticular formation, which divides the liver parenchyma into many hexagonal structures with similar morphology and functions, called hepatic lobules.^76^ The hepatic lobule is the basic structure of the liver, with a height of about 2 mm and a width of about 1 mm. The liver parenchyma of a normal adult is composed of over 500,000 hepatic lobules. In the center of the hepatic lobule is the central vein (CV), surrounded by the portal area, which contains the branches of the hepatic artery, PV, and hepatic duct. Blood flows from branches of the hepatic artery and PV to the hepatic sinusoids, where it undergoes material exchange with hepatocytes into the CV, while bile flows from the center to the portal area of the hepatic lobules. Bile is eventually pooled into a network of bile ducts, independent of the blood system.^77^ Many organs exhibit spatial heterogeneity along tissue depth and the longitudinal axis of blood vessels, and the liver is a good example of this heterogeneity. In the hepatic lobule, from the portal area to the central venous area, the metabolic activity and functional expression of the liver change gradually, which may be related to hormone, oxygen concentration gradients, and signaling pathways.^78^, ^79^ Hepatocytes in the portal zone have higher activities of gluconeogenesis, ammonia detoxification, and ureagenesis, whereas hepatocytes and those in the central venous zone have higher activities of glycolysis and phase 1 drug metabolism.^78^, ^80^

Liver sinusoids, the lacunae between adjacent liver plates, are specialized capillaries that are porous and slightly narrower than blood cells.^81^ The liver has approximately 1 billion hepatic sinusoids, which provide a wide area for exchange of substances between hepatocytes and blood. Generally, fenestrae on the liver sinusoids allow the transfer of various substances. However, when they are senescent or cirrhotic, the appearance of pseudocapillarization and capillarization hinders this mass transfer.^82^, ^83^ The space around the sinusoids is known as Disse's space. It is a small gap of about 1.4 μm between liver sinusoidal endothelial cells (LSECs) and hepatocytes. The microvilli of the hepatocytes around the sinusoids stretch or extend beyond Disse's space and come into direct contact with the blood.

**FIGURE 2:**
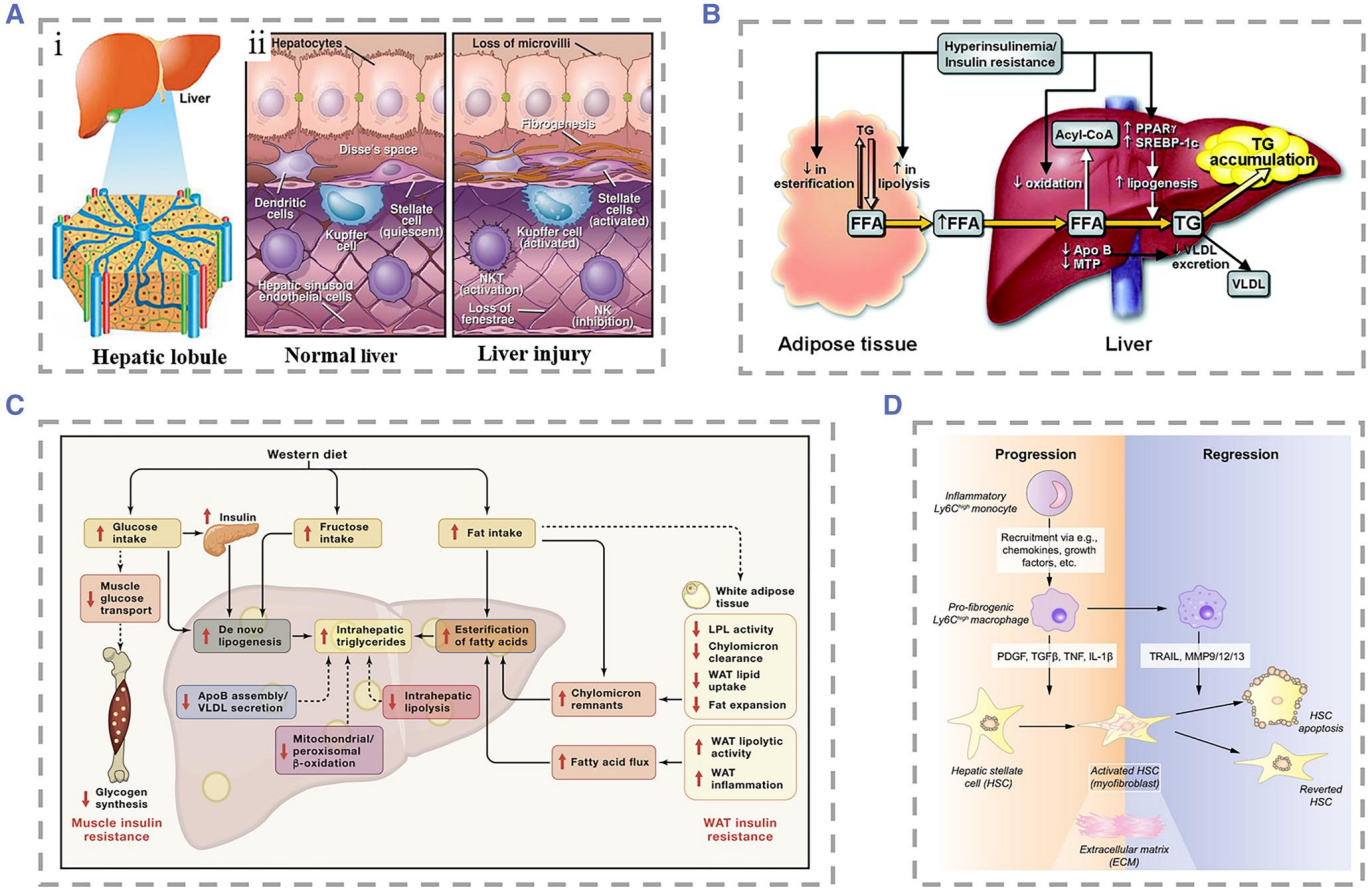
(A) (i) Gross view of the liver and schematic representation of hepatic lobules. Reproduced under terms of the CC‐BY license.^69^ Copyright 2019, The Authors, published by Multidisciplinary Digital Publishing Institute. (ii) Localization of various classes of cells in normal and injured liver. Reproduced with permission.^98^ Copyright 2013, John Wiley and Sons. (B) Pathogenesis of nonalcoholic hepatic steatosis. Reproduced with permission.^99^ Copyright 2005, Can Med Assoc. (C) Metabolic causes of NAFLD. Reproduced with permission.^100^ Copyright 2021, Elsevier. (D) Interactions between macrophages and stellate cells in the progression of liver fibrosis. Reproduced with permission.^101^ Copyright 2015, Elsevier.

The liver mainly consists of four types of cells. They can be divided into parenchymal cells and non‐parenchymal cells (NPCs). Parenchymal cells are hepatic cells (HCs). NPCs include hepatic stellate cells (HSCs), LSECs, and Kupffer cells (KCs). The liver parenchyma is composed of hepatocytes, accounting for 65% of the total cells in the liver. Hepatocytes are usually polyhedral in shape and have a size of about 20–30 μm.^84^, ^85^ The cytoplasm contains a large number of organelles such as mitochondria, endoplasmic reticulum, and lysosome, which are related to the ability of material metabolism and protein synthesis. Almost all albumin is synthesized by hepatocytes, which produce about 12 g per day, accounting for 25% of the total protein produced by the liver.^86^ The hepatocytes from the portal area to the CV are arranged in a plate shape and closely connected with each other. In nonalcoholic fatty liver (NAFL), triglyceride accumulates in hepatocytes and causes hepatic steatosis, which eventually leads to the death of hepatocytes.^87^ Normal HSCs are spindle‐shaped, located mainly in the subendothelial space between the hepatocytes and LSECs.^88^, ^89^ The most typical feature of HSCs is that they can store fat droplets and retinoids in the cytoplasm.^90^ After liver injury, HSCs are activated to secrete extracellular matrix (ECM) under the stimulation of cytokines, which contributes to the structural reconstruction of the liver.^89^ Meanwhile, they differentiate into contractile myofibroblasts, which is associated with the progression of fibrotic liver disease. LSECs constitute the wall of the sinusoid. Due to the lack of cell‐to‐cell junctions between the LSECs, many 100 nm‐sized fenestrae are formed on the wall of the sinusoid, allowing only particles smaller than the diameter of the fenestrae to pass through.^91^ Therefore, LSECs can be regarded as the “selective sieve” for material exchange between hepatocytes and sinusoid blood. LSECs can selectively endocytose a variety of substances relying on the endocytic vesicles in their cytoplasm as well as remove macromolecular metabolic waste from the systemic circulation.^92^, ^93^, ^94^, ^95^, ^96^ KCs are macrophages, making up 80%–90% of human tissue macrophages.^97^ They reside in the sinusoids and are responsible for phagocytosis and clearance of pathogenic microorganisms from the gastrointestinal tract, immunoreactive substances that pass through the sinusoids, and senescent red cells.^97^ Activated KCs contribute to the damage process of hepatocytes by releasing cytokines, superoxides, and other bioactive substances (Figure 2A).^69^, ^98^

At any moment, the liver contains 14% of the body's total blood volume. The liver not only clears harmful substances from portal blood but also drains the blood stored in hepatic sinusoids to maintain blood volume when the body loses blood. Toxins in human blood and antigenic substances of pathogenic microorganisms are mostly detoxified and eliminated in the liver. For example, ammonia can be converted into urea in the liver and excreted through urine, heavy metal ions are eliminated through the bile into the intestinal lumen and excreted through feces.^102^, ^103^ The liver is also the site of coagulation factor synthesis, such as factor II, VII, IX, and X, which can stanch when blood vessels are ruptured by trauma or other causes.^104^, ^105^ Another well‐known function of the liver is to promote digestion by secreting bile.^106^ The hepatocytes synthesize bile, and the bile ducts connect the lobules to the larger ducts leading to the bile ducts. Bile is transported into the gallbladder and finally drains into the duodenal lumen stimulated by food. The release of bile contributes to the intestinal digestion and absorption of fats and fat‐soluble vitamins.^107^

### 2.2 Pathology

Primary liver cancer mainly includes three pathological types: hepatocellular carcinoma (HCC), intrahepatic cholangiocarcinoma (ICC), and mixed HCC‐ICC.^108^, ^109^ Hepatocellular carcinogenesis can be caused by viral interference, the presence of chronic liver disease, and exposure to chemicals. Premalignant lesions of HCC usually include dysplasia of hepatocytes, low‐grade dysplastic nodules, and high‐grade dysplastic nodules, etc.^110^ It can be observed that the cells exhibit morphological changes such as increased or decreased volume, deepened nuclear staining, and multinucleation. Early stage HCC exhibits local structural abnormalities and high cell density in the liver, but the cellular atypia is often not evident.^111^ Unfortunately, the diagnosis of HCC is usually made after patients have developed certain clinical symptoms and liver impairment. At this time, HCC has often progressed to an advanced stage, and effective therapies for prolonging survival are lacking.^112^, ^113^ Therefore, reading up on the liver cancer pathological changes at the early stage and exploring high accuracy and low‐cost screening methods can help to diagnose liver cancer early and improve the prognosis of patients. The liver‐on‐chip platform described here is an emerging approach to study the pathological processes of liver cancer.^114^, ^115^, ^116^, ^117^, ^118^

The association of hepatic steatosis with obesity, diabetes, and alcohol has been demonstrated at a very early age.^119^ Obese patients and diabetics have a higher risk of developing NAFLD compared with the normal population.^120^, ^121^, ^122^ Insulin resistance leads to hyperinsulinemia, which increases serum free fatty acid levels.^123^ The liver absorbs excess free fatty acids and drives triglyceride production and the development of steatosis (Figure 2B).^99^ As the condition worsens, the histological changes can manifest as simple steatosis, steatohepatitis, and fatty fibrosis, and eventually progressing to cirrhosis. Steatosis in NAFLD is usually macrovesicular and occurs first in the central venous zone, where the cytoplasm of hepatocytes contains a single large fat droplet and results in displacing the nucleus to the periphery of the cytoplasm. In addition to steatosis, histological changes such as lobular inflammation and lipogranulomas are also present in NAFLD. An important characteristic change in nonalcoholic steatohepatitis (NASH) is hepatocyte ballooning, which causes the swelling of hepatocytes with pale cytoplasmic staining and hyperchromatic nuclei with prominent nucleoli.^124^, ^125^ Apoptotic bodies and lytic necrosis can also be observed. Lobular inflammation and portal inflammation are usually mild, but the combination with other liver diseases should be considered when the severity of portal inflammation does not correspond to the degree of hepatocellular lesion (Figure 2C).^100^ Hepatic fibrosis usually firstly occurs in the lobule 3 region of the liver and is mainly characterized by the accumulation of ECM. HSCs predominate in the progression of liver fibrosis because they can be activated by stimulatory factors after liver injury and subsequently produce large amounts of ECM.^126^ Activated macrophages produced stimulatory factors: transforming growth factor *β* (TGF‐*β*) and platelet‐derived growth factor, among others. In addition, PV myofibroblasts and bone marrow‐derived cells are also involved in liver fibrosis as NAFLD progresses.^127^ Viral hepatitis tends to cause bridging fibrosis with bridging necrosis and fibrous septa in the CV.^128^ Liver fibrosis is initially reversible; however, it can progress to irreversible cirrhosis with complications, such as portal hypertension, and jaundice and finally lead to death or waiting for liver transplantation (Figure 2D).^101^, ^129^, ^130^

The highly complex architecture of the liver, especially the microarchitecture and the need for precise expression in engineered systems, make it well suited to apply microtechnology to fabricate liver‐on‐chips. A comprehensive understanding of liver microstructure and physiology is crucial to help to build the biomimetic liver‐on‐chips. Besides, based on the chips, it can be realized that using the in vitro models to study the physiopathological changes and microstructural alterations caused by liver disease.

## 3 CULTURE ENVIRONMENT OF LIVER‐ON‐CHIPS

In order to simulate the microenvironment of the liver in the human body, the researchers have explored many methods, and the construction of in vitro liver‐on‐chips is one of the important ones. Liver‐on‐chips technology can more realistically reproduce the microenvironment of the liver and thus, it shows obvious superiority in the drug toxicity test and the liver disease mechanism research. To meet different needs, researchers have developed different types of liver‐on‐chips.^131^, ^132^, ^133^ Herein, we will summarize the culture environment of the different liver‐on‐chips.

### 3.1 2D liver‐on‐chips

**FIGURE 3:**
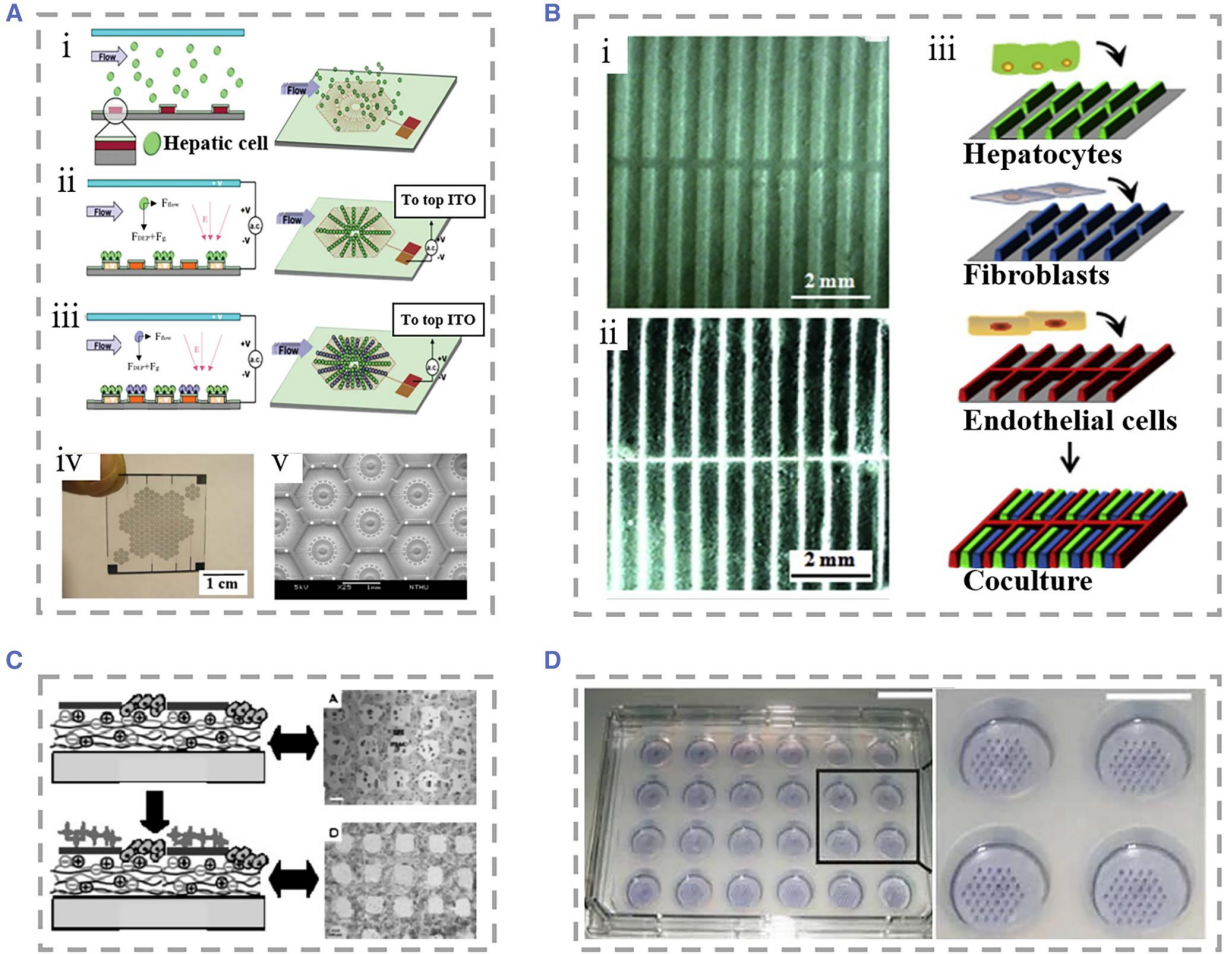
(A) (i–iii) Diagram of the steps used to manipulate cells to form specific patterns using dielectrophoresis force. Hepatocytes were randomly seeded in microfluidic chambers. (iv and v) The formed patterned co‐culture chips. Reproduced with permission.^141^ Copyright 2013, The Royal Society of Chemistry. (B) (i and ii) SEM morphologies of patterned fibrin mats with ridge/sulcus widths of 200/300 and 100/400 μm. (iii) Schematic of co‐culture of hepatocytes with fibroblasts and ECS on the electrospinning system. Reproduced with permission.^142^ Copyright 2016, Elsevier. (C) Schematic and physical illustration of the formation of hepatocyte and fibroblast patterned co‐cultures on the surface of polyelectrolyte multilayers. Reproduced with permission.^143^ Copyright 2007, John Wiley and Sons. (D) Photograph and local magnification of a 24‐well apparatus with micropatterned co‐cultures. Reproduced with permission.^136^ Copyright 2008, Springer Nature.

Traditional dish‐based culture techniques for hepatocytes are difficult to show cell–cell interactions and cell–matrix interactions and simulate the complex liver microenvironment due to its limitations, of which different kinds of hepatocytes are mixed and seeded randomly.^134^ To overcome this limitation, a novel approach co‐cultures two or more cell types in a specific order and position through cell patterning techniques, thereby coordinating heterotypic cell interactions and mimicking the true distribution profile of liver cells.^135^ Some strategies were used to improve the precision of cell patterning,^134^, ^136^, ^137^ such as Hannachi and coworkers, who used the microcontact printing technique to fabricate micropatterned co‐cultured cell sheets, and Nahmas et al., who used laser‐guided direct writing technique to draw cell patterns with high accuracy.^138^, ^139^ In addition to passive patterning, dielectrophoresis (DEP), a method of actively manipulating cellular patterning, is widely used.^140^ Ho et al. manipulated positive DEP forces that attract randomly scattered cells within the culture chamber to fix in the area of maximum electric field, thus creating a specific pattern that mimics the structure of liver lobules (Figure 3A).^141^ After two days of culture, they found that the activity of CYP450 enzymes in patterned HepG2 cells was 80% higher than that in nonpatterned HepG2 cells. Liu et al. proposed an electrospinning technique to construct micropatterned collectors on glass substrates.^142^ They loaded hepatocytes, fibroblasts, and endothelial cells into the electrospinning system, and seeded these cells into patterned mats, thereby establishing a patterned co‐culture platform (Figure 3B). The activity of enzymes, such as CYP3A11, reached the highest level on day 5 of culture, and maintained high activity for up to 15 days. Kidambi et al. fabricated a liver‐on‐chip by taking advantage of the characteristic that hepatocytes preferentially attached to sulfonated polycystene (SPS).^143^ They used microcontact printing technology to form SPS patterns on polyelectrolyte multilayer (PEM). Hepatocytes would preferentially attach to the SPS region and subsequently covered up the fibroblasts to form a patterned co‐culture (Figure 3C).

In addition to the above methods, researchers have also presented a variety of cell co‐culture methods based on 2D patterning. Macdonald and coworkers used paper substrates to culture patterned cells, which allowed the seamless transfer of cellular tissue into target culture plates.^144^ Zhang et al. reported an inkjet printing technology applied to liver‐on‐chip, which helped to improve the efficiency of hepatocyte patterning.^145^ The micropatterned method can be applied to the testing of drug toxicity and the infection of hepatitis viruses. Khetani et al. combined soft lithography and microtechnology to adhere hepatocytes and collagen domains on a polydimethylsiloxane (PDMS) template before overlaying mouse fibroblasts to form a micropatterned co‐culture (Figure 3D).^136^ The balance of intercellular interactions is regulated by adjusting the diameter of the channel in the template. They demonstrated the effect of co‐culture by assessing gene expression profiles, secretion of liver specific products, and susceptibility to hepatotoxins. In Ploss's work, they reproduced the full life cycle of the whole hepatitis C virus under a multi‐well format.^146^

### 3.2 Matrix‐free 3D liver‐on‐chips

**FIGURE 4:**
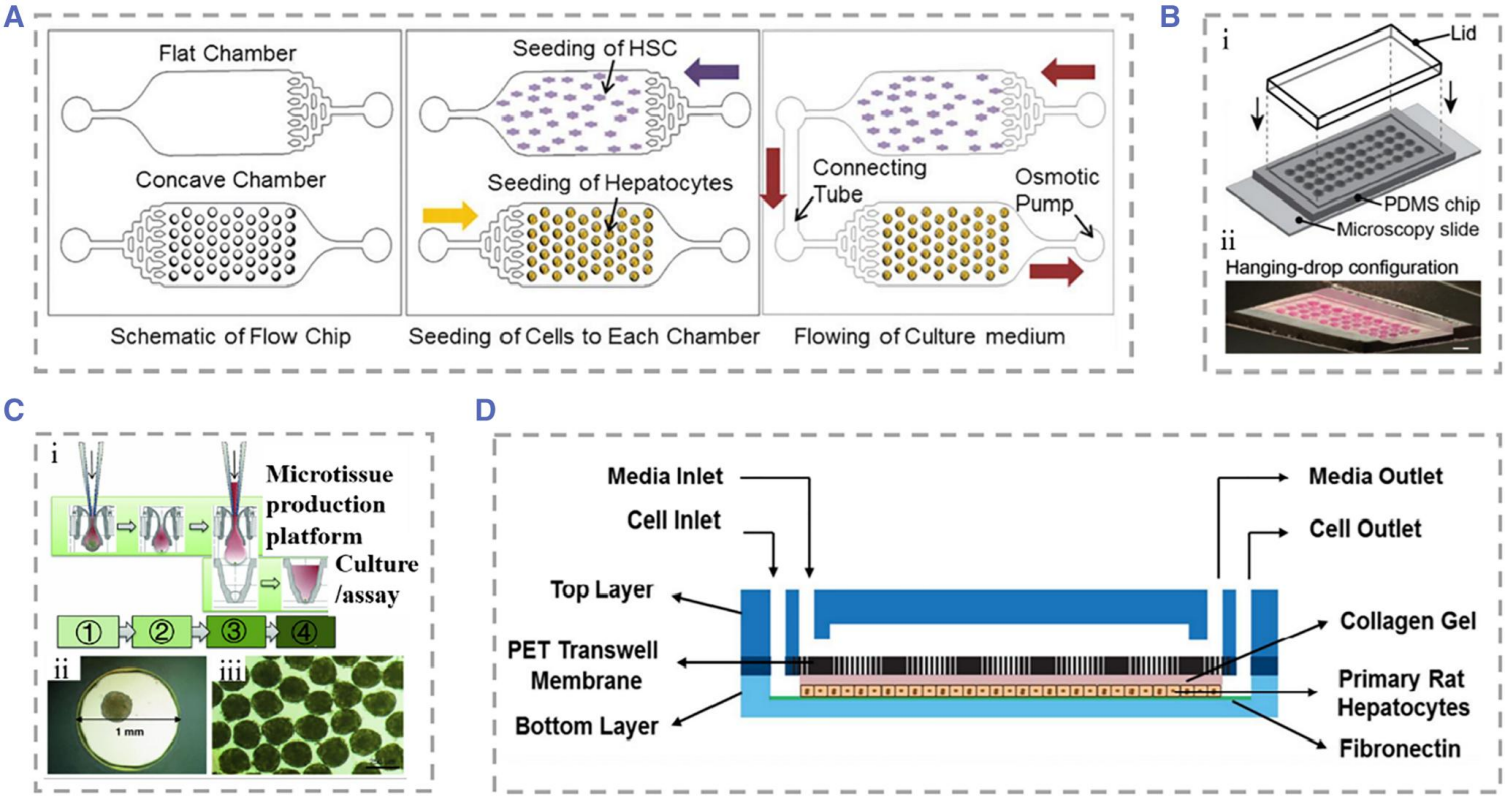
(A) The liver‐on‐chip consisted of a flat chamber with cultured HSCs and a concave chamber with cultured hepatocyte spheroids and a connecting tube between the two chambers. Reproduced with permission.^152^ Copyright 2013, The Royal Society of Chemistry. (B) (i) Design diagram of the microfluidic hanging drop platform. The microfluidic network was patterned on the surface of PDMS. (ii and iii) I chip is run in both hanging drops. Reproduced under terms of the CC‐BY license.^153^ Copyright 2019, The Authors, published by John Wiley and Sons. (C) Schematic representation of liver mammosphere production and culture. (i) Microsphere formation was followed by transfer to gravity PLUS™ for further culture and analysis. ① Cell Seeding, ② Microtissue Maturation, ③ Microtissue Transfer, ④ Microtissue Culture/Assay. (ii and iii) Bright field microscopy of microspheres from human liver. Reproduced under terms of the CC‐BY license.^154^ Copyright 2013, The Authors, published by Springer Nature. (D) Schematic diagram of the microfluidic device. The medium flowed from the top layer, and hepatocytes were cultured in the bottom layer. Reproduced with permission.^155^ Copyright 2014, The Royal Society of Chemistry. HSC, hepatic stellate cell; PDMS, polydimethylsiloxane.

Despite the unique advantages of 2D liver‐on‐chips, there is still an obvious gap in simulating the in vivo microenvironment in comparison with 3D culture.^147^ 3D culture of hepatocytes facilitates the maintenance of cellular morphological stability and function, and is beneficial for quantitatively understanding cell–cell interaction effects under a simulated environment.^148^, ^149^, ^150^, ^151^ In order to investigate the interaction between hepatocytes and HSCs and to distinguish between direct cell contact and paracrine effects on hepatocytes, Lee and his colleagues innovatively proposed a meniscal‐based method to rapidly and simply construct 3D liver‐on‐chips with size‐controllable hepatocyte spheroids.^152^ The chip consisted of a flat chamber to culture HSCs and a concave chamber to culture hepatocyte spheroids. The two chambers were connected using a connecting tube with a Polyethylene glycol (PEG) solution flowing from the stellate cell medium to the hepatocyte spheroid, which mimicked the velocity of in vivo intercellular interstitial flow (Figure 4A). Hepatocyte spheroids cultured in flow medium showed a significant increase in survival and exhibited the highest functional activity at day 8 compared to liver‐on‐chips lacking medium flow. It was also investigated that hepatocyte spheroids were optimally maintained at the PEG solution flow rate of 5.53 mm/h.

The hanging drop technique in the absence of the matrix is another approach to cluster cells into 3D spheres. The hanging drop technique has advantages in achieving in situ formation, growth of cells, as well as diffusion of metabolites. Boos and coworkers used this technique to propose a microfluidic device consisting of two culture chambers and a channel in the middle that could be blocked by partition, which were incubated with hanging drops carrying human liver microtissues (hLiMTs) and embryonic bodies (EBs), respectively (Figure 4B).^153^ Using gravity pumps to facilitate communication between the two, they found that metabolites of hLiMTs could be transferred into EBs and have potentially adverse effects on their growth. Messner et al. also used the hanging drop technique to create a 96‐well format plate specifically designed to manufacture hanging drops of hepatocytes mixed with NPCs.^154^ The cultured hepatocyte globules had stable structure and good activity (Figure 4C). Wong et al. developed a concave microwell‐based culture platform to create spheroids of controllable size self‐aggregated from primary hepatocytes and HSCs, demonstrating the role of HSCs in stabilizing spheroids.^156^ In addition to the above‐mentioned methods, Miyamoto et al. used the Tapered Stencil for Cluster Culture device for the large‐scale preparation of uniform‐sized spheroid‐based 3D liver‐on‐chips, effectively addressing the problem that uneven sizes of spheroids formed by common culture methods cause difficulties in experimental reproduction.^157^

### 3.3 Matrix‐dependent 3D liver‐on‐chips

In liver‐on‐chips engineering, how to prolong the survival time of hepatocytes and maintain liver function in vitro is still a challenge to be addressed.^158^ Achieving optimal liver function relies on the interaction not only between hepatocytes and NPCs but also the cell and matrix.^159^ In recent years, many natural or synthetic ECM components have been applied in in vitro liver models.^160^, ^161^, ^162^, ^163^, ^164^ Natural source components include proteins (including but not limited to collagen and silk fibroin), sugars (chitosan and alginate), polylactic acid and other derivatives, and synthetic components including PEG and others.^165^, ^166^ ECM has a proven effect in promoting cell adhesion, maintaining cell survival, and promoting cell–matrix interactions. For example, Kim et al. conducted studies on primary rat hepatocytes (RPHs) and found that embedding rat hepatocytes into PEG‐heparin hydrogels incorporating hepatocyte growth factor (HGF) could keep hepatocytes well bioactive after 3 weeks of culture.^167^ Fan et al. added galactosylated hyaluronic acid (GHA) to a chitosan (CS) scaffold.^168^ Relative to the chitosan scaffold, CS/GHA resulted in improved albumin secretion and stabilized cytochrome P450 activity in primary mouse hepatocytes. They then added heparin to the CS/GHA scaffold using a similar approach.^169^ The microenvironment for hepatocyte growth was improved by taking advantage of the high affinity of HGF to bind heparin.

Because human primary hepatocytes are difficult to obtain and culture, researchers commonly use HepG2 cells as an alternative to perform matrix‐dependent 3D liver‐on‐chip studies. For example, Lang and coworkers extracted ECM from porcine liver to provide a culture platform for the seeding of human HepG2 cells after removal of immune substances, maintaining cell function for at least 3 weeks of culture time.^170^ Toh's team presented a cell culture system based on 3D microfluidic channels with the aim of enabling precise control of cell–matrix interactions.^171^ They selected a collagen and then formed a thin layer matrix of cultured HepG2 cells through the process of polyelectrolyte complex coacervation. This system could provide hepatocytes with a 3D growth morphology and perfusate flow environment, which was beneficial for maintaining the physiological structure of hepatocytes and for quantitatively collecting data on the occurrence of cellular events during culture by integrating different elements for the system. In this method, the matrix was coated on the surface of the cell culture. In another strategy, the cells are embedded in a gel containing the ECM. Hedge and coworkers described a “sandwich” structural culture platform by encapsulating hepatocytes in two layers of collagen, and the top layer added with flowing culture fluid (Figure 4D).^155^ They found that hepatocytes had good activity under flow conditions and showed a bile duct‐like junction between hepatocytes after 2 weeks of culture. Gieseck III et al. report a method to promote the maturation of induced pluripotent stem cell‐derived hepatocytes (iPSC‐Heps).^172^ Collagen type I fibrillogenesis induced by heating encapsulated iPSC‐Heps, followed by water removal to form collagen hydrogels of physiological collagen density and phenotypic alterations of cells toward primary hepatocytes can be clearly found. Skardal et al. developed a new type of hydrogel using the liver‐specific semisynthetic ECMs, which was prepared by mixing the extract of acellular liver ECM or whole liver tissue with collagen Type I and hyaluronic acid.^173^

## 4 CELL SYSTEM CLASSIFICATION

The liver is an organ with a complex structure and function, and people adopt microfluidic chips with different cell culture systems, aiming to mimic the microenvironment of the liver as precisely as possible in vitro. The cell culture system of the liver‐on‐chip is becoming increasingly complex, from single‐type cell culture, multi‐cell co‐cultures, to multi‐organ collection systems.^69^, ^174^, ^175^

### 4.1 Single‐type cell culture

**FIGURE 5:**
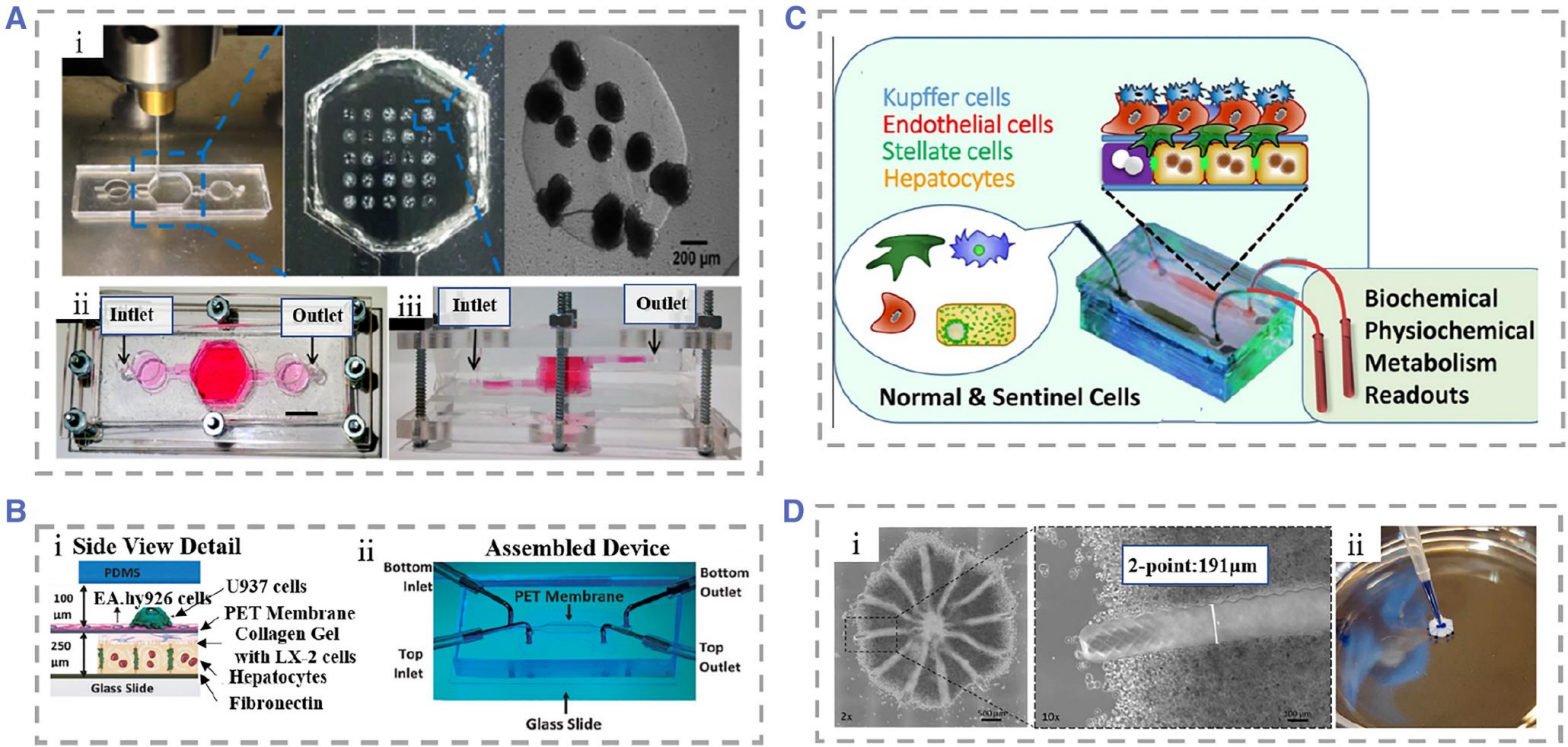
(A) (i) The hydrogel structure was bioprinted on polymethylmethacrylate using a laser cutting mold. (ii and iii) The top‐view and side‐view of the assembled liver‐on‐chip. Reproduced with permission.^182^ Copyright 2016, IOP Publishing. (B) (i) Detailed side view of the device with four kinds of cells cultured in layers to mimic the microenvironment of liver sinusoids. (ii) Assembled microfluidic device. Reproduced with permission.^183^ Copyright 2016, John Wiley and Sons. (C) The liver acinus module with a microchip, including a diagram of four liver cell types. Reproduced with permission.^184^ Copyright 2013, Springer Nature. (D) (i) Microscopic picture of the printed liver model, and the hollow duct structure is visualized at 10× magnification. (ii) Channel structures are demonstrated to be hollow using trypan blue dye. Reproduced under terms of the CC‐BY license.^185^ Copyright 2018, The Authors, published by Multidisciplinary Digital Publishing Institute.

In microfluidic chips, the most frequently adopted cell culture system is single‐type cell microspheres because it is well established, inexpensive, and easily reproducible, and therefore it is widely used in drug toxicity testing and cell metabolism models.^176^, ^177^, ^178^, ^179^, ^180^ Kizawa et al. designed a scaffold‐free spheroid of hepatocytes that did not contain any ECM or NPCs.^181^ They used the hepatocyte spheroids to investigate bile acid secretion and used insulin to inhibit the camp/protein kinase A pathway to reduce glucose production in hepatocyte spheroids. Bhise et al. encapsulated HepG2/C3A cells in photocrosslinkable gelatin methacryloyl (GelMA) hydrogels.^182^ They used bioprinting to rapidly fabricate large quantities of liver models and were able to control the thickness of the hydrogel outer layer of the encapsulated hepatocytes (Figure 5A). Their 3D liver structures could preserve function for 30 days and apply long‐term toxicity assessment. To explore whether primary human hepatocytes cultured in vitro have the ability to restore liver tissue‐like cellular structures and form bile canalicular networks, Goral and coworkers proposed a device for primary human hepatocyte culture without natural or artificial ECM.^148^ Under the condition of perfusion, the hepatocytes were tightly bound to each other to form a tissue‐like structure for at least 2 weeks. They also found that the added fluorescein diacetate was taken up by hepatocytes and transported into the formed bile canalicular structures.

In vivo, hepatocytes are polyhedral cells with tight junctions between each other, but when hepatocytes are isolated and cultured in vitro, many characteristic features disappear as hepatocytes spreading on culture plates.^186^ Due to the lack of support from NPCs, with the gradual increase of culture time, the nuclei of hepatocytes undergo karyolysis, cell boundaries become blurred, and the due cell activity was lost. In summary, although the single‐type cell culture system is simple to construct and has been applied to many aspects such as biomedicine and disease diagnosis, the short survival time of hepatocytes, the lack of interaction between different cells, and the disappearance of tissue‐specific structures limit further development.^187^

### 4.2 Multiple type cell co‐culture

There are complex interactions and interactions between two or more types of cells in the liver, so single‐type cell culture is not enough to mimic the real liver microenvironment. Co‐culture of hepatocytes with NPCs has been shown to maintain hepatocyte morphology and a variety of liver functions, because NPC populations have important roles in metabolizing transforming drugs, detoxifying, and modulating hepatocyte metabolic competence. Therefore, the adding of one or more types of NPCs enables the chip more liver functions.^188^, ^189^, ^190^ For example, SCs remodel the phenotype of the liver ECM through myofibroblasts, KCs play an important role in the liver's response to injury through the production of cytokines and reactive oxygen species, and LSECs are involved in the liver's powerful regenerative capacity. Therefore, the investigators co‐cultured hepatocytes and NPCs at a ratio similar to that of the native liver that enabled reproduction of the liver microenvironment in vitro.^191^ Bhatia et al. used soft lithography to micropattern hepatocytes and fibroblasts.^192^ By controlling the adhesion area of cells on the culture plate, the ratio of hepatocytes to supporting cells could be precisely controlled. They explored the optimal ratio to achieve the maximization of effectiveness in NPCs, providing a reference for future related research. Compared to static cell culture, microfluidic systems can better mimic the in vivo tissue situation. Prodanov et al. studied a new type of micro flow control device.^183^ They put hepatocytes, EA.hy926, LX‐2, and U937 cells into the equipment once to make the experimental device with a multi‐layer structure. The fluid in the device cannot be applied directly to hepatocytes but must pass through the endothelial monolayer formed by U937 cells. This mimicked the real liver sinusoidal microenvironment as accurately as possible (Figure 5B). Similarly, Vernett I and coworkers chose hepatocytes, EA.hy926, LX‐2, and U937 cells as cell sources.^193^ These SQL‐SAL models could survive at least 28 days under flowing conditions. Bhushan et al. established a 3D human liver‐on‐chips mimicking acinus, and their strategy was to seed hepatocytes first, followed by the sequential addition of NPCs.^184^ The cells will be proportionally distributed at appropriate physiological ratios of 70%–80% hepatocytes, 15%–20% endothelial cells, 5%–10% stellate cells, and 5%–10% Kupffer cells (Figure 5C). Their system also includes a database from which information derived from liver chips will be used to aid in the interpretation of microphysiological readouts and the development of computational models. However, the creation of this sandwich cell ECM layer increases the difficulties of manufacturing and integration.

Some researchers have selected human‐induced pluripotent stem cells (hiPSCs) as their study subject. They proposed that hiPSCs‐derived hepatocyte‐like cells (HLCs) remain immature.^17^, ^194^, ^195^ Ma et al. suggested that the imperfect function of HLCs resulted from the lack of support from NPCs during hiPSCs differentiation.^196^ Thus, they reported a digital light processing (DLP)‐based liver‐on‐chip model in which they placed hiPSC derived hepatic progenitor cells (hiPSC‐HPCs) in and co‐cultured with umbilical vein endothelial cells and adipose‐derived stem cells in hydrogels. Compared with traditional 2D single hiPSC cultures, the co‐cultured multiple type cells exhibited significant improvements in protein synthesis function and cytochrome p450 activity. Ahmed et al. innovatively used a modified polyethersulfone hollow fiber (HF) membrane under static and dynamic conditions, respectively, to establish an in vitro liver model by seeding primary human sinusoidal endothelial cells, stellate cells, and hepatocytes on the HF membrane sequentially.^197^ Stellate cells produce growth factors that promote hepatocyte growth, and at the same time, hepatocytes secrete insulin‐like growth factor I to maintain the activity of stellate cells. Thanks to interactions between hepatocytes, sinusoidal cells, and stellate cells, the model maintained high levels of the protein synthesis function and the biotransformation function of diazepam for 28 days. To address the issue of restricted diffusion of oxygen and nutrients in spheroid culture, Grix and coworkers employed bioprinting technology to seed hepatocytes and stellate cells onto a printed liver model to mimic human liver lobules.^185^ There were 12 channels from the edges to the central port in the model, which allowed the perfusion of the culture fluid to the entire structure. The printed liver tissue system maintained higher bioactivity of over a 2‐week culture period (Figure 5D).

### 4.3 Multi‐organ collection system

**FIGURE 6:**
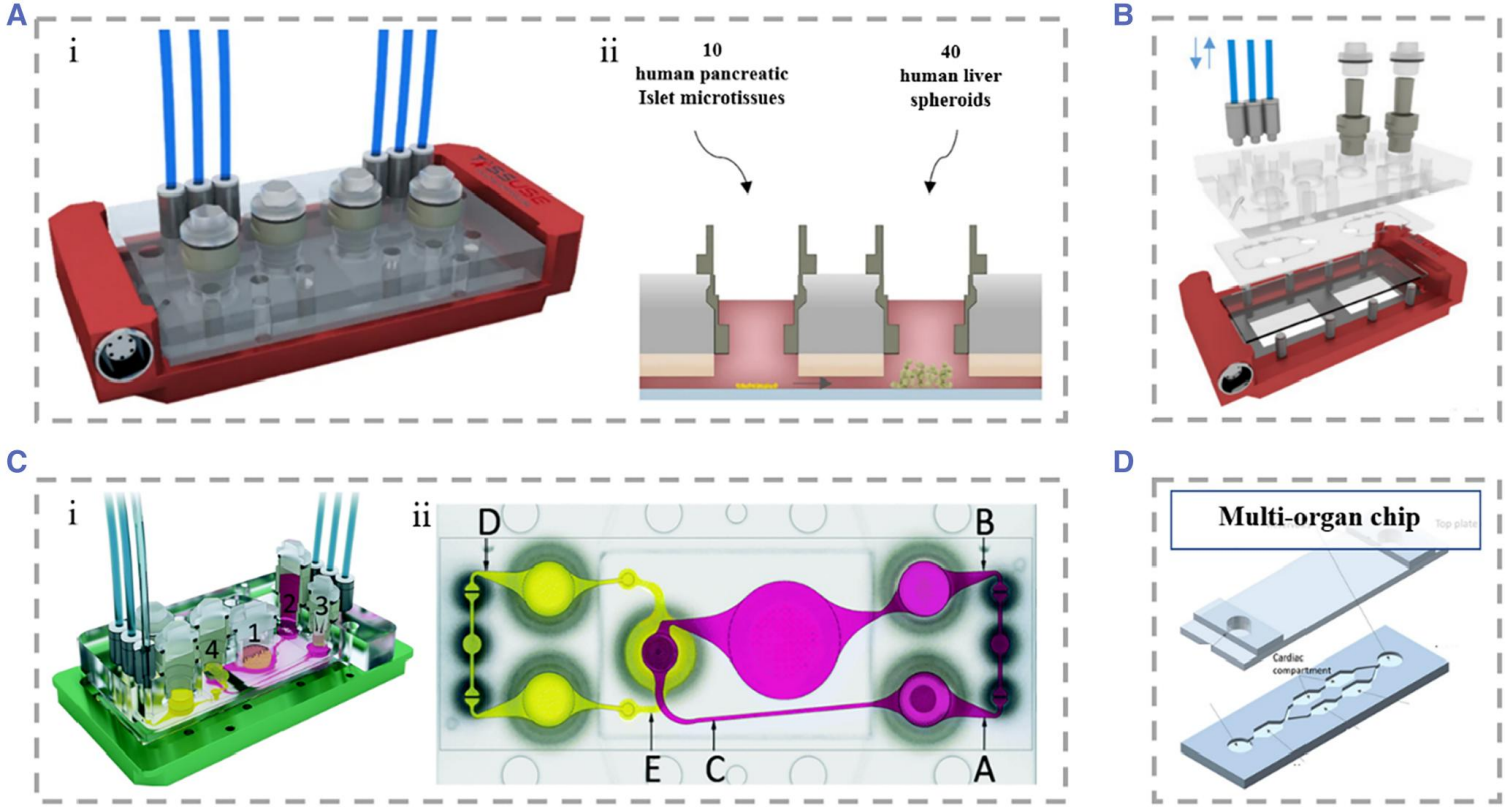
(A) Dual‐organ chip of liver and pancreatic islets. (i) 3D view of the assembled device. (ii) Loading scheme for organ equivalents of liver and pancreatic islets. The two chambers communicate with each other. Reproduced under terms of the CC‐BY license.^208^ Copyright 2017, The Authors, published by Springer Nature. (B) Schematic representation of the multi‐organ chip system allowing co‐culture of three tissues. Reproduced with permission.^209^ Copyright 2015, Elsevier. (C) (i) Schematic of the device for the four‐organ chip. Numbers represent gut (1), liver (2), skin (3), and kidney (4) tissue microarrays. Alternative flow circuits (pink) and excretory flow circuits (yellow). (ii) Schematic of the measurement points of the blood flow circuit (A, B, and C) and the measurement points of the excretion circuit (D and E). Reproduced under terms of the CC‐BY license.^210^ Copyright 2015, The Authors, published by the Royal Society of Chemistry. (D) Schematic representation of the microfluidic chip containing multiple chambers. Different cells were segmented into different chambers, and material exchange was achieved between chambers relying on gravity. Reproduced under terms of the CC‐BY license.^211^ Copyright 2016, The Authors, published by Springer Nature.

The various organs within the human body are not isolated, and the complete process of drug metabolism and the normal life activities of humans require the joint participation of multiple organs. Therefore, it is difficult for a single‐organ chip to represent the physiological functions of all the systems in the human body.^63^, ^198^, ^199^ Many scholars load multiple organ chips on a single microfluidic device to explore the connection between individual organs.^200^, ^201^, ^202^, ^203^, ^204^, ^205^, ^206^, ^207^ For example, Bauer et al. co‐cultured liver tissue and islet tissue in an insulin‐free microfluidic device to explore the link between the liver and pancreas, two key organs for maintaining glucose homeostasis (Figure 6A).^208^ It is known that insulin released by pancreatic islet b cells promotes glucose uptake by hepatocytes and conversion into hepatic glycogen for storage, whereas insulin resistance in the liver of patients with type 2 diabetes mellitus (T2DM) leads to poor glycemic control. In their work, islet tissue co‐cultured with liver tissue over a 15‐day period produced more stable levels of insulin compared to islet tissue culture alone. The co‐cultured liver tissue reduced the glucose level in the medium to that of normal human postprandial blood glucose within 48 h. Meanwhile, the liver tissue reduced glucose uptake capacity after 3 days of culture, which might relate to the production of insulin resistance by the hepatocytes. This multi‐organ chip will provide a reference for the mechanism of T2DM progression.

Most of the multi‐organ chips had a very large volume of circulating medium. In order to solve this problem, Materne et al. constructed a multi‐organ chip‐based system, which consisted of a chip of two independent microfluidic circuits and high 100 μm channels connecting the two circuits.^209^ Each circuit contained a 96‐well plate on which liver tissue and skin punch biopsies were seeded separately. The entire microfluidic channel circuit was covered by human dermal microvascular endothelial cells (Figure 6B). The multi‐organ chip maintained the activity of the three tissues for up to 28 days under flow cultured conditions and could be used for drug metabolism studies. Their system supported a maximum of three tissue co‐cultures and was therefore underpowered to predict true systemic response to drugs. To observe the complete process of drug absorption, distribution, metabolism, and excretion, Maschmeyer et al. combined four human organ chips, intestine, skin, liver, and kidney, and connected them using a microfluidic device.^210^ They planted primary human small intestinal epithelial cells at the top layer of the device to provide a barrier function between the outside of the intestine and the device as well as the absorption process of substances. The absorbed substances that entered the surrogate blood circuit were transported to a liver‐like structure composed of HepaRG and stellate cells, where the material was further metabolized by the simulated liver. The metabolites would enter the excretory circuit through the PET membrane, and human proximal tubular cell line RPTEC/TERT‐1 cultured on the PET membrane mimicked the excretory function of the kidney. Finally, skin biopsy could be used for tests of metabolite toxicity (Figure 6C). Oleaga et al. produced another type of multi‐organ chip containing four types of cells: heart, liver, skeletal muscle, and neurons.^211^ Different tissues were segmented into different chambers, and material exchange between individual chambers was achieved by gravity driven flow (Figure 6D). This system maintained for up to 14 days, which opened up the possibility for chronic toxicity testing of drugs. They tested doxorubicin, atorvastatin, valproic acid, and acetaminophen on the chip for drug toxicity, providing a viable research tool for future drug toxicity studies.

## 5 APPLICATIONS

Since they simulate the tissue structure and function of the liver, liver‐on‐chips hold great promise for liver transplant donor substitution, drug toxicity experiments, metabolic monitoring, and evaluation.^212^, ^213^, ^214^, ^215^

### 5.1 Bioartificial liver and liver transplantation

**FIGURE 7:**
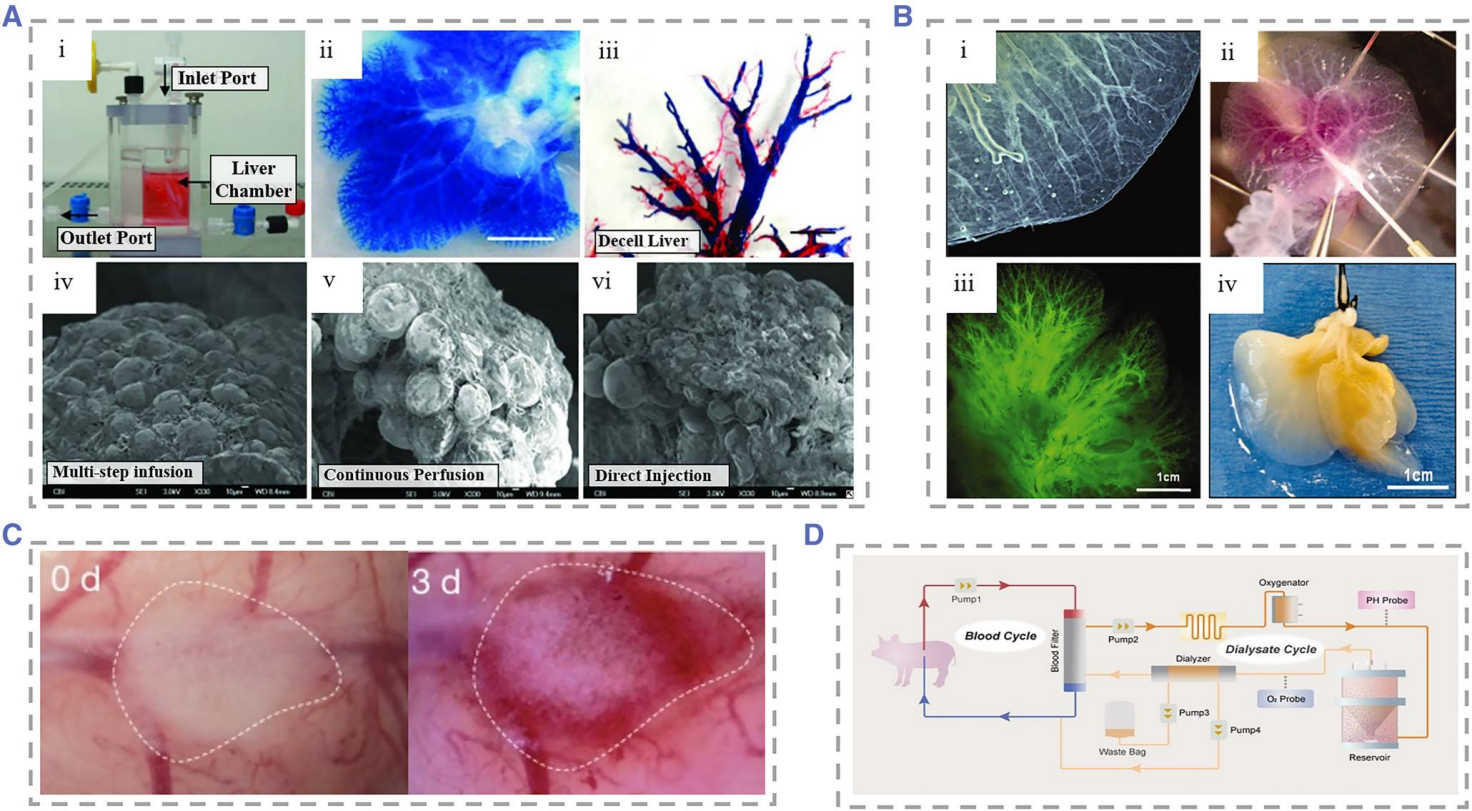
(A) (i) Physical diagram of customized organ chamber. The decellularized liver matrix was placed in the main chamber of the device. (ii) Microvascular tree perfused with blue dye. (iii) Corrosion cast model of the decellularized liver. PV (blue), artery (red), vein (yellow), and bile duct (green). (iv)–(vi) SEM micrographs of recellularized liver after 4 days of culture using three cell seeding techniques. Reproduced with permission.^224^ Copyright 2011, Mary Ann Liebert, Inc. (B) (i) Photograph of a decellularized liver lobe. The liver parenchyma, liver capsule, and vasculature can be clearly visualized. (ii) Injection of fluorescein‐labeled 250 kDa dextran into the PV of decellularized mouse livers revealed vessel patency. (iii) Fluorescence microscopy of the decellularized liver of (ii), showing the liver native vascular tree. (iv) Photograph of the right lobe of the ferret liver 7 days after cells were seeded. Recellularization of the liver parenchyma was seen. Reproduced with permission.^225^ Copyright 2011, John Wiley and Sons. (C) Generation process in the human liver with a functional vascular network. Reproduced with permission.^226^ Copyright 2013, Springer Nature. (D) Schematic diagram of the spherical reservoir bioartificial liver device. After blood was drawn from the animal, it passed through a blood filter composed of hollow fiber channels. Sphere reservoir the hepatocyte spheroids are stored. Red and blue lines indicate the blood compartment and orange lines indicate the decellularized albumin dialyzate compartment. Reproduced with permission.^227^ Copyright 2017, AlphaMed Press.

For patients with liver failure, liver transplantation may be the only treatment option, but the current scarcity of donor livers cannot meet the increasing demand.^216^ To alleviate the donor shortage problem of liver transplantation, researchers actively construct various in vitro artificial liver support systems to provide temporary liver function support for patients with liver failure and strive for time to wait for the donor liver.^217^, ^218^, ^219^ Shi et al. developed an artificial liver system based on a multilayer plate bioreactor, which successfully rescued canines suffering from acute liver failure.^220^ In another study, they transplanted Human functional hepatocytes based on the extracorporeal cell‐based bioartificial liver into pigs with liver failure.^221^ This was the first reported artificial liver system utilizing stem cell‐derived HLCs. The system could not only detect liver specific secretions but also improve survival of pigs with statistical significance. Unfortunately, discordance in HLCs differentiation and maturation, confers a higher risk of tumorigenesis after transplantation compared with Primary human hepatocytes (PHHs). This hinders the application of HLCs as artificial livers.^222^, ^223^ Soto‐Gutierrez et al. proposed a whole organ liver decellularization method with minimal disruption, which preserved the ultrastructure of the liver, the intact vascular network, and bile drainage system.^224^ The decellularized livers were recellularized afterward using three different reseeding methods, and the multistep strategy resulted in the greatest seeding efficiency and survival rate of functional hepatocytes (Figure 7A). This work provided a new approach for constructing a transplantable liver.

To meet the demand for clinical use, it requires approximately 10^10^ hepatocytes to fill the recellularized liver scaffolds. Filling so many hepatocytes into the scaffold was difficult to achieve because of the lack of intrafolded channels.^228^ To solve this problem, Baptista et al. developed decellularized liver scaffolds with the removal of liver parenchyma and preservation of the vascular skeleton (Figure 7B).^225^ The aim of their study was to make it easy for human hepatocytes to enter the scaffold and fill the scaffold volume, thus providing a highly efficient method for the fabrication of donor liver graft substitutes. Since the source of human primary hepatocytes is quite limited and liver cancer cells have decreased function and a potential risk of carcinogenesis, it is necessary to explore the possibility of generating liver donors from hiPSCs. Takebe et al. constructed functionalized and vascularized surrogate livers using hiPSCs in the absence of donor liver replacement.^226^ Hepatocytes generated from hiPSCs, mesenchymal stem cells (MSCs), and human umbilical vein endothelial cells (HUVECs) were co‐cultured and formed explants, which could connect to host vessels within 48 h and show patency, and the vascular network formed by induced pluripotent stem cell‐liver buds (iPSC‐LBs) was similar to that in a normal adult liver (Figure 7C). It was observed that iPSC‐derived tissue could produce albumin approximately 10 days after transplantation and maintained a high level of secretory function thereafter. In order to improve the survival rate of hepatocytes after recellularization, Yap et al. implanted hepatic progenitor cells in spheroids.^229^ Compared with conventional single‐cell suspensions, this strategy improved cell survival rate and proliferation. In another study, it was proposed that the liver‐derived hMSCs (LHMSCs) have functions such as proliferation, regulation of immune response, and secretion of trophic factors.^230^ They found that LHMSCs have potential for further development in liver regeneration and liver transplantation. Currently, most of the bioartificial livers remain in the preclinical experimental stage, and although there have been promising results in the application of artificial livers in pigs, the application of these therapeutic strategies in clinical treatment must also overcome the problems, such as insufficient cell numbers, safety of the cells, and so on (Figure 7D).^227^

### 5.2 Drug toxicity experiments

**FIGURE 8:**
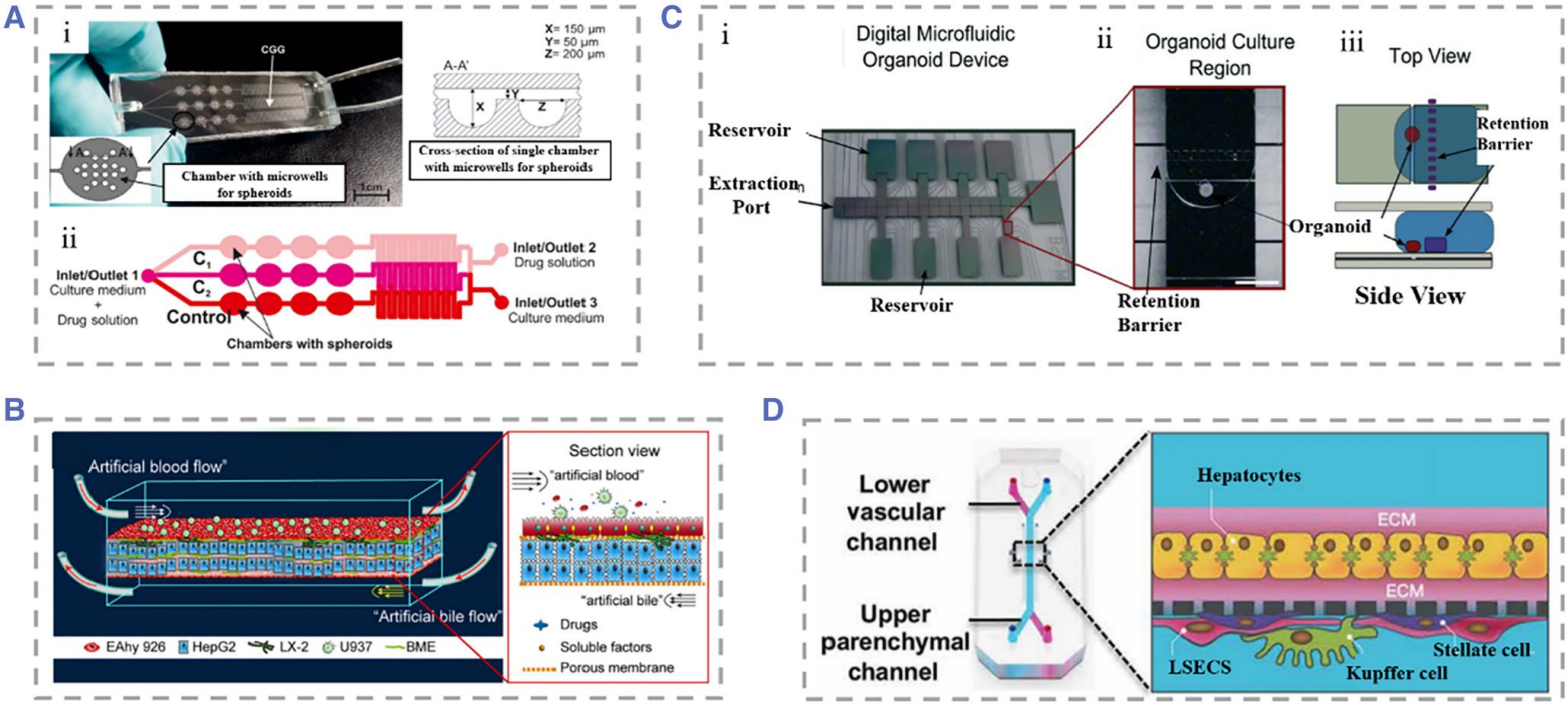
(A) (i) Physical illustration of the microfluidic system‐based 3D hepatocyte spheroid culture platform. (ii) The drug solution was distributed in different concentrations within the device. Reproduced with permission.^236^ Copyright 2017, John Wiley and Sons. (B) Schematic representation of the species and distribution of the four types of cells seeded in the device. Reproduced with permission.^237^ Copyright 2019, AIP Publishing. (C) (i) Physical diagram of microfluidic organoids for drug screening equipment without moving parts and valveless fluid manipulation. (ii) Organoid culture region, defined by a retention barrier. (iii) Top and side views of the device. Reproduced with permission.^238^ Copyright 2014, The Royal Society of Chemistry. (D) Schematic of the rat, dog, and human liver‐on‐chip. Primary hepatocytes were grown in the upper parenchymal channel, with the upper and lower sides coated with ECM. NPCs (LSECs, KCs, and SCs) were seeded in the lower vascular passage. Reproduced with permission.^239^ Copyright 2019, The American Association for the Advancement of Science.

There has been extensive academic research aimed at exploring in vitro models of hepatotoxicity of therapeutic drugs.^16^, ^231^, ^232^, ^233^, ^234^ The liver‐on‐chip model accurately mimics the in vivo liver microstructure and dynamic flow environment. Meanwhile the drug perfusion time and concentration can be precisely controlled, which helps scholars achieve a balance between drug toxicity and efficacy.^235^ Besides that, compared to animal organs and thick tissue sections, the liver‐on‐chip is friendly for imaging and analysis due to its advantages in volume and can be used to monitor cell status in real time. The liver‐on‐chip can also make up a multi‐organ chip with other organ chips to explore the whole process of drugs from absorption to metabolism.^233^ Zuchowska et al. established a microfluidic system based on HepG2 spheroid to analyze the effectiveness of the anticancer drug 5‐fluorouracil (5‐FU) by varying the concentration of 5‐FU and the size of hepatoma cell spheroids.^236^ They found that the resistance rate of HepG2 to 5‐FU decreased as the spheroids grew larger (Figure 8A). However, single hepatocytes are less metabolically competent due to the lack of cell–cell interactions, which limits the role in hepatotoxicity assessment. Deng et al. used a novel strategy to create a physiological mimetic microenvironment.^237^ They established a liver sinusoidal chip composed of four types of cells (HepG2 cells, LX‐2 cells, Eahy926 cells, and U937 cells) in which hepatic blood flow channels and bile efflux channels flowed in opposite directions parallel to each other. They used the chip to test acetaminophen hepatotoxicity and interactions of acetaminophen with other drugs (Figure 8B). Vernetti et al. used the same cell source to test apoptosis and ROS after troglitazone, nimesulide, and trovastatin exposure via a fluorescent protein biosensor.^193^ Ma et al. designed a liver‐on‐chip composed of a radial hepatic myeloid network and liver sinusoidal network, which maintained high basal CYP‐1A1/2 and UGT activities.^240^ They used this chip to investigate potential adverse drug interactions of acetaminophen, isoniazid, and rifampicin, and provide a feasible platform for in vitro toxicology studies.

Innovations to the liver‐on‐chip in recent years have been made for not only more accurate simulation of the liver microenvironment but also the simpler and more applicable device to drug toxicity testing, or extended to multispecies liver‐on‐chip, to observe the relevance of induced hepatotoxicity in animals to humans. Au et al. proposed the strategy of “fail early, fail cheaply.”^238^ They established a hepatic organoids chip by co‐culturing HepG2 and NIH‐3T3 cells. The device achieved the generation and culture of mixtures without loading moving parts and minipumps. They used three metrics: construct contractility, viability, and albumin production to characterize liver organoids (LOs). Moderate hydrogel shrinkage helped the study of cell densities to approach those of native tissue; most cells were still alive and had higher albumin secretion at 4 days of culture. Through control of these metrics, they demonstrated that the device can form and maintain viable LOs. Monitoring the effect of dilution series of acetaminophen on hepatocyte activity by assays for CYP enzyme activity and hepatotoxicity demonstrates the applicability of their research applications to drug screening. This device could be a cost‐effective tool in drug research and development (Figure 8C). To better distinguish the different hepatotoxicity phenotypes, Jang et al. designed a liver‐on‐chip including human, dog, and rat.^239^ Two‐ or four‐cell co‐cultures were performed under physiological fluid flow to observe the different hepatotoxic phenotypes such as hepatocyte damage and steatosis that appeared in the chip (Figure 8D). The chip can be used to analyze the correlation of drug‐induced animal hepatotoxicity with human hepatotoxicity and elaborated the mechanism of action of human hepatotoxicity.

Single‐organ chips focus on mimicking the function of this organ, while multi‐organ chips strive to describe the metabolism and transport processes of drugs across different organs. Mello et al. proposed a heart–liver–skin three‐organ chip system.^232^ In this system, the effects of drug exposure on heart and liver function were assessed by adding different drugs and using both topical administration and systemic application. The chip could effectively predict the acute and chronic toxicity of drugs, and skin surrogate could be used to test drug toxicity via transdermal drug delivery. In another attempt, Baert et al. established a liver–testis organ chip system by placing the primary adult testicular cells and hepatocytes in separate culture chambers on a chip.^206^ When the medium was supplemented with anti‐neoplastic prodrug cyclophosphamide, the rising levels of specific cytochromes within hepatocytes and germ cell death were observed.

### 5.3 Metabolic monitoring and assessment

**FIGURE 9:**
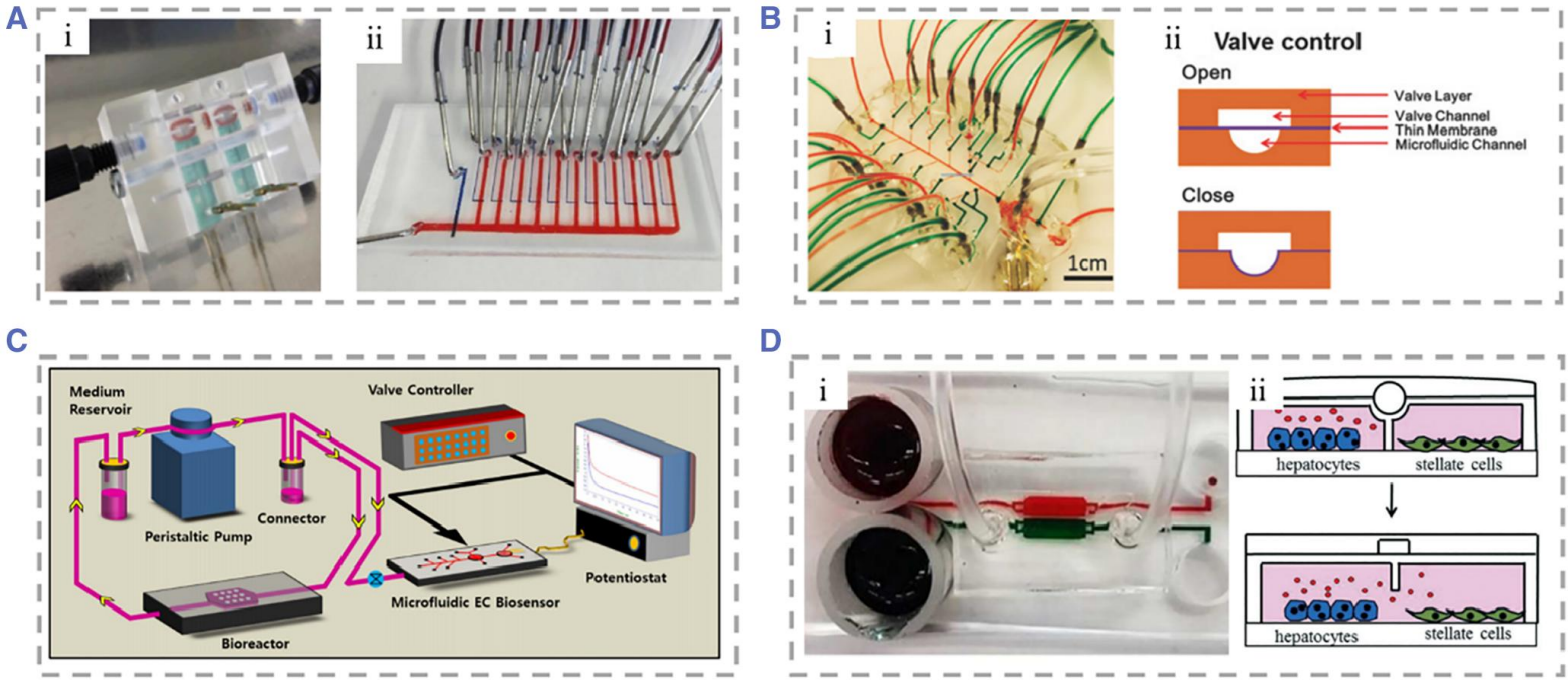
(A) (i and ii) Photograph of the polymethylmethacrylate device containing glucose and lactate sensors. Reproduced with permission.^247^ Copyright 2016, National Academy Sciences. (B) (i) Microfluidic EC biosensors that can be used for fully automated biosensing measurements. (ii) The switching of the valve is controlled by gas‐actuated microvalves. Reproduced under terms of the CC‐BY license.^248^ Copyright 2017, The Authors, published by John Wiley and Sons. (C) Schematic of bioreactor detection using the EC biosensor. Reproduced under terms of the CC‐BY license.^249^ Copyright 2016, The Authors, published by Springer Nature. (D) (i and ii) Real and cut views of a typical microfluidic device for co‐culture experiments to observe changes after alcohol damage. Reproduced with permission.^250^ Copyright 2015, The Royal Society of Chemistry.

Tracking the activity of tissues and organs is essential in the studies of drug toxicity and efficacy.^241^ However, most organ‐on‐chips rely on endpoint assays to assess drug toxicity and mitochondrial activity, resulting in a lack of real‐time monitoring of viability and metabolic changes to the tissue organ that provides limited dynamic information.^242^ Therefore, there is an increasing need to improve current models and develop accurate, efficient, and long‐term monitoring liver‐on‐a‐chip biosensing platforms.^78^, ^243^, ^244^, ^245^ At present, a variety of physical, electrochemical, and optical sensors are used in organoids to continuously monitor the metabolic activity and secretory function of the cells. Combining biosensors with microfluidic chip technologies will promote the efficiency of drug toxicity evaluation and reduce the cost of drug research and development.^246^ Bavli et al. proposed a liver‐on‐a‐chip that can maintain tissue activity under physiological conditions for more than 1 month.^247^ They made an off‐chip sensor and all used air purification before measurement to record changes in glucose metabolism from oxidative phosphorylation to anaerobic glycolysis with real‐time monitoring of mitochondrial respiration. They also added twelve self‐addressable micromechanical valves to the chip to enable automated execution of experimental steps (Figure 9A). Based on this chip, the kinetics of cellular adaptation to mitochondrial damage induced by rotenone and troglitazone was described, and it was found that troglitazone caused mitochondrial damage at concentrations previously considered safe. In order to reduce manual operation and improve the automation of the sensor work. Shin et al. innovatively developed a unique liver‐on‐chip applying electrochemical (EC) biosensor on which a microfluidic valve allowed regeneration and detection with full automation.^248^ Sensors utilizing electrochemical impedance spectroscopy measurements for fully automated monitoring of cell secreted biomarkers including human albumin and glutathione enable long‐term monitoring of cellular metabolic activities (Figure 9B). Riahi et al. constructed a liver‐on‐chip combined with a magnetic microbeads (MBs)‐based EC immunosensor.^249^ Disposable MBs could stay on the chip with an applied magnetic field to immobilize biomarkers, whereas they can be flushed out of the chip and collected after the magnetic field is withdrawn. The chip could assay hepatocellular metabolism over a long period of time by detecting cellular secretions such as transferrin and albumin (Figure 9C). Zhou et al. constructed a bioreactor for the co‐culture of hepatocytes and stellate cells.^250^ Paracrine crosstalk between the two cell types was monitored by examining the secretion profile of TGF‐β provoked by alcohol injury using the three miniature aptamer‐modified electrodes placed in separate chambers. Partitions separating the chambers in the device are movable and they can move and change the configuration of the device and the contact ways of the two cells. They compared three models of alcoholic liver disease (ALD) and found that alcohol damage causes hepatocytes to secrete TGF‐β, diffuse to surrounding stellate cells, and activate them to produce more TGF‐β (Figure 9D).

**FIGURE 10:**
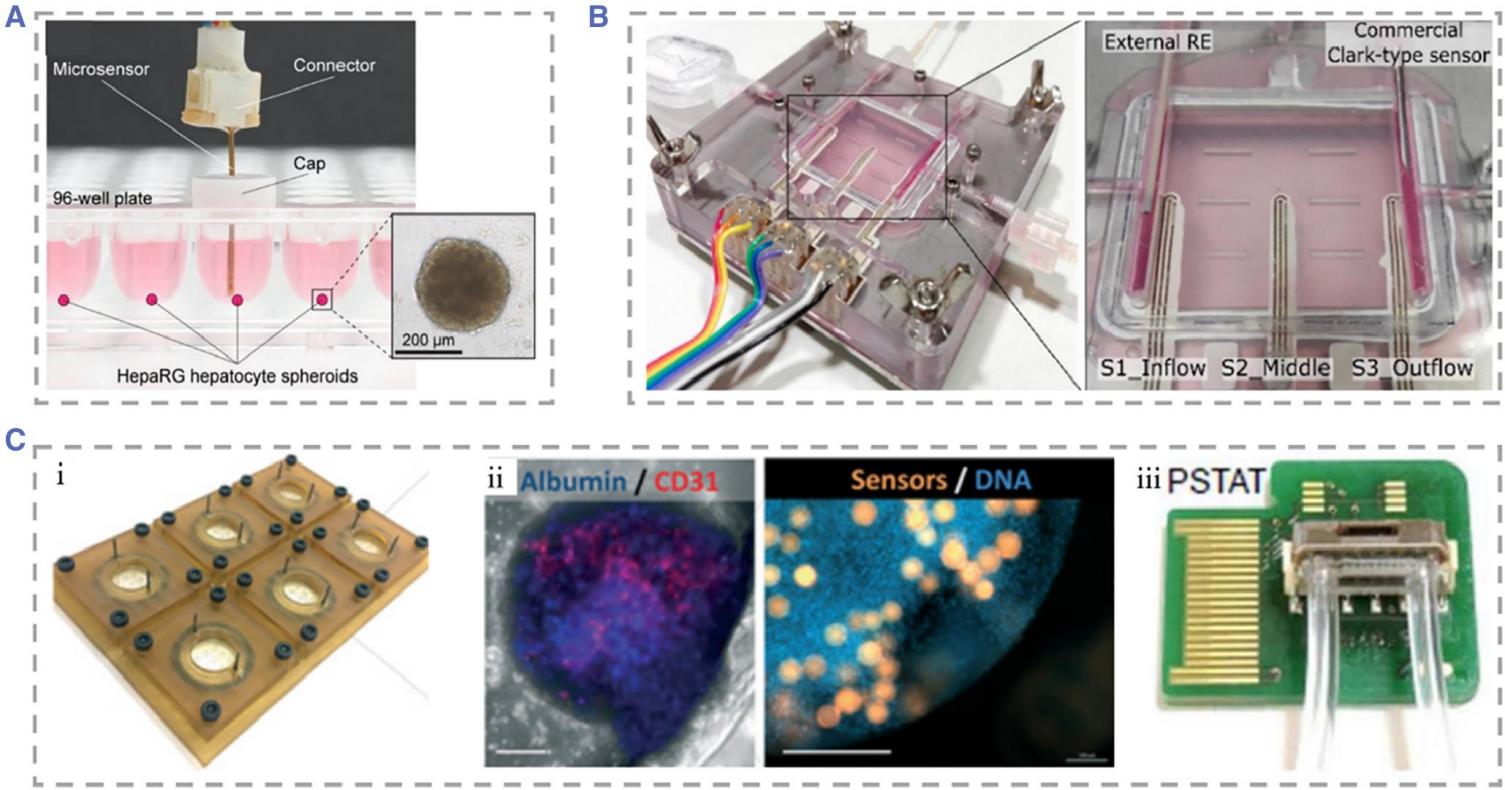
(A) Physical image of a 96‐well cell culture plate. Reproduced with permission.^251^ Copyright 2017, Elsevier. (B) Optical image of the Rennert's chip. Reproduced with permission.^243^ Copyright 2018, The Royal Society of Chemistry. (C) (i) Schematic representation of the 6‐well bioreactor plate. (ii) Immunofluorescence staining of hepatocytes and endothelial cells. (iii) Photograph of a microfluidic chip integrated with a temperature sensor and an on‐chip potentiostat. Reproduced with permission.^252^ Copyright 2018, The Royal Society of Chemistry.

Weltin et al. achieved convenient, precise, and long‐term detection of cellular metabolism by integrating EC sensors of lactate and oxygen in the liver‐on‐chip.^251^ With lactate levels in media starting at 50 µM with a production rate of 5 μM h^−1^, the sensor accurately quantified the amount and timing of lactate produced by spheroids while monitoring hypoxia in microwells. They observed that the addition of the hepatotoxic drug bosentan caused a dose‐dependent decrease in lactate production by the cells (Figure 10A). To solve the problem of sensor integration difficulties, Moya et al. successfully embedded them into culture membranes without affecting their filtration function.^243^ They applied inkjet printing technology on the liver‐on‐chip to form uniform sensor devices in the target regions for real‐time monitoring of oxygen concentrations in human and rat hepatocytes (Figure 10B). They used 96‐well cell culture plates and applied the sensor to rapidly assay metabolic parameters of cultures inserted directly into the wells of titration plates. Rennert et al. designed a microfluidic liver‐on‐a‐chip co‐cultured HepaRG, HUVEC, monocyte macrophage, and human stellate cell line LX‐2. A 34 × 28.5 mm cell culture area was located in the center of the device, with the exterior accessible for connections to a variety of sensors and functional elements.^253^ Oxygen luminescence‐based sensors were integrated at the import and export of the chip, allowing real‐time monitoring of cellular oxygen consumption.

There have also been study controlling concentration gradients of added metabolic modulators to investigate the separate effects of each modulator and the controlled metabolic patterns of hepatocytes, showing zonation of hepatocyte glucose metabolism, ureagenesis, and drug metabolism. Ehrlich et al. designed a liver‐on‐chip integrated with sensors.^252^ The embedded microprobes were used to dynamically monitor oxygen concentration, while an EC sensor was used to monitor glucose, lactate, and temperature. Immunofluorescence staining showed albumin positive hepatocytes (blue) and CD31 positive endothelial cells (red). The oxygen sensor (orange) is embedded in the microtissue (blue). They used this chip to track the metabolic changes in the liver exposed to the epilepsy drug valproate and the antiretroviral drug stavudine and found a related mechanism of disrupted cellular metabolic homeostasis and drug‐induced liver injury at the drug concentrations previously considered safe (Figure 10C).

### 5.4 Establishment of liver disease models

The development of in vitro models that mimic the pathophysiology of the liver in vivo is one of the important topics in liver‐on‐a‐chip studies and will help elucidate disease mechanisms and treatments. A series of liver disease models have been established, including liver cancer, NAFLD, ALD, viral hepatitis, and patient‐specific liver disease.^254^, ^255^, ^256^

#### 5.4.1 Liver cancer organoid chip

**FIGURE 11:**
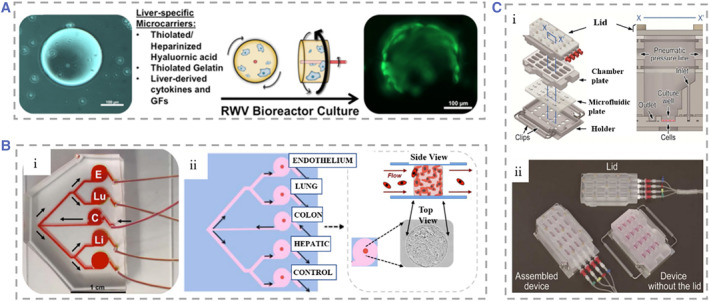
(A) Schematic diagram of liver tumor spheroid formation process in RWV. Hepatocytes gradually adhered to form spheres around microcarriers in RWV. Reproduced with permission.^264^ Copyright 2017, IOP Publishing. (B) (i–iii) Design of organoid multi‐organ chip platform and biofabrication of orthotopic tumors. Reproduced with permission.^265^ Copyright 2019, John Wiley and Sons. (C) (i) Schematic of the culture equipment based on pneumatic pressure‐driven medium system containing 16 chambers, and the structures inside the culture chamber at x‐x′ cross sections. (ii) Fabricated physical picture of multi‐organ microfluidic equipment. Reproduced with permission.^266^ Copyright 2018, The Royal Society of Chemistry. RWV, rotational wall vessel.

An in vitro 3D liver tumor model is a reliable strategy for studying the mechanism of tumor spread and drug screening.^116^, ^257^, ^258^, ^259^, ^260^, ^261^, ^262^ Skardal et al. established a liver cancer model in a rotating wall vascular bioreactor.^263^ The rotational wall vessel (RWV) is an in vitro suspension culture system. HepG2 cells and HCT‐116 metastatic colon carcinoma cells were added to RWV at the ratio of 10:1, and the microgravity state generated from the rotation promoted the aggregation of the cells on the microcarrier beads, generating liver tissue containing colon carcinoma tumor foci. This model could serve as a platform to observe changes in tumor cells in real time. Devarasetty and coworkers have also used RWV production to make host‐liver colorectal‐tumor spheroids, which could be used for observing the growth of tumor cells and researching anticancer drugs (Figure 11A).^264^ Skardal and coworkers explored a circulating fluid device to explore metastasis of tumor cells.^267^ The device contained two separate chambers to culture intestinal and hepatic tissues, respectively, and I two chambers were connected in series by circulating fluid flow to simulate tumor metastasis. It was observed that HCT‐116 cells could grow continuously, break through the intestinal structures and successfully invade the liver structures in the chip. They also set up a liver‐on‐chip consisting of an upstream chamber containing cancer tissue and multiple downstream chambers containing tissues from different organs to evaluate the tumor's preferential metastatic propensity.^265^ After two bifurcations, perfusion from a single inlet I into the device provided the same amount of perfusion to the chambers where different tissues were located. The cells in the chambers realized the interaction and material exchange with cancer cells through recycling media (Figure 11B). In another attempt, Wang et al. proposed that a microfluidic chip with multilayered polymethylmethacrylate (PMMA) and PDMS mimicked intrahepatic renal cancer cell progression.^116^ They plated kidney cancer cells (Caki‐1) and hepatocytes (HpeLL) on decellularized liver matrix/GelMA. The ratio of the two types of cells was changed to mimic the degree of invasion of renal cancer cells in the liver to evaluate the efficacy of anticancer drugs 5‐FU at different ratios. Satoh et al. reported a multi‐organ‐on‐chip model with pneumatic pressure driven media circulation.^266^ The model could be used for two organ systems and four organ systems by employing different microfluidic plates (Figure 11C). Based on this device, liver and colon cancer models were employed to investigate the inhibitory effect of 5‐fluorouracil (5‐FU), a metabolite of the anticancer prodrug capecitabine (CAP) on cancer cells.

#### 5.4.2 Nonalcoholic fatty liver disease chip

NAFLD refers to the excessive deposition of fat in the liver that caused excluding excessive alcohol and other clear pathogenic factors. To date, researchers have not defined the mechanism of NAFLD. NAFLD can be classified into NAFL, NASH, and NASH‐related cirrhosis based on the degree of pathologic change and whether hepatic fibrosis occurs.^268^ Rodent models of NAFLD are widely used, but there are major differences between humans and animal models, such as the mechanisms of fat accumulation, the degree of liver fibrosis, and so on. In vitro 3D models based on human liver cells are therefore promising research directions.^269^, ^270^ To better understand and explore NAFLD, it is important to develop accurate, low‐cost, and long‐term culturable in vitro models.^271^, ^272^

**FIGURE 12:**
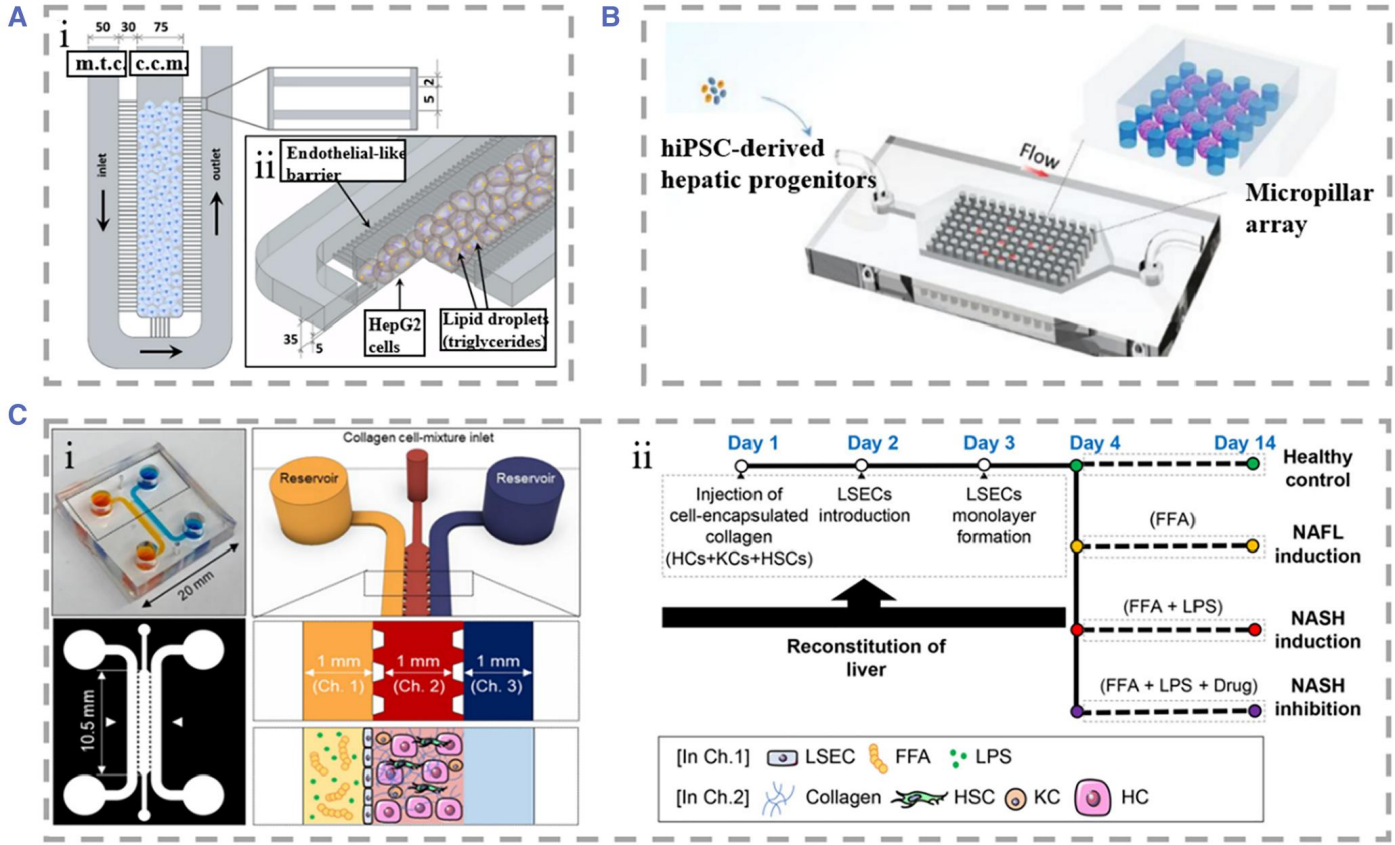
(A) Microstructure of the NAFLD‐on‐chip. Top view (i) and 3D schematic (ii) of the device. Reproduced under terms of the CC‐BY license.^275^ Copyright 2016, The Authors, published by the Public Library of Science. (B) (i) NAFLD is a progressive liver disease characterized by simple steatosis, inflammation, and metabolic abnormalities. The liver may progress to fatty livers under conditions of excessive FFA exposure. (ii) Design of the NAFLD on chip. (iii) Step of generating steatohepatitis from HiPSCs on chip. Reproduced with permission.^256^ Copyright 2020, American Chemical Society. (C) (i) Physical diagram of the microfluidic NAFLD‐on‐chip. The central channel of the model contained a mixture of HCS, KCs, HSCs, and hydrogels, and the channel was surrounded by inlet and outlet channels. (ii) Time frame of reconstruction of the liver‐on‐chip and various stages of NAFLD. Reproduced with permission.^276^ Copyright 2021, The Authors, published by John Wiley and Sons. Ch., channel; FFA, free fatty acid; HCS, hepatic cell; HiPSCs, human‐induced pluripotent stem cells; HSC, hepatic stellate cells; KCs, Kupffer cells; NAFLD, nonalcoholic fatty liver disease.

Giraudi et al. established a hepatocytes and HSCs co‐culture model in which the two kinds of cells are closely connected.^273^ In this system, they found that HSCs play a key role in initiating the progression of NAFLD‐induced liver fibrosis. 2D co‐culture models are one of the important tools to explore the mechanisms of free fatty acids (FFAs)‐induced inflammation in NAFLD. However, 3D liver models can better mimic the microarchitecture of the liver. Some studies constructed 3D spheroids of human hepatocytes, HUVECs, and KCs co‐cultured to show the different stages of steatosis. Nevertheless, the lack of dynamic perfusion caused lower oxygen, nutrient delivery, and metabolite clearance, which ultimately affected the activity of hepatocyte spheroids.^274^ Gori et al. developed a 3D liver model for studying the pathogenesis of NAFLD (Figure 12A).^275^ At the center of the device was a cell culture microchamber (c.c.m.), and hepatocytes were cultured at high density inside the chamber. Mass transport channels (m.t.c.) surrounded the chamber periphery. The channel communicated with the chamber to enable transport of nutrients and clearance of metabolites. They chose palmitic and oleic acids, the most abundant FFAs in the human diet, to add into the culture medium to simulate the pathogenesis of NAFLD. Wang and coworkers established an in vitro disease model of NAFLD based on hiPSC.^256^ The system contained a microcolumn array structure, which made the hiPSC‐derived hepatic progenitor cells form the same micromass size. Meanwhile, the microfluidic channels in the device could realize perfusion of nutrient solution and drainage of metabolic waste (Figure 12B). With these designs, they achieved stable formation and long‐term culture of LOs. Under the induction of free fatty acids (FFAs) for 7 days, obvious lipid droplets could be seen in LOs, indicating the occurrence of steatosis in hepatocytes. This system provided a reference for learning the mechanism of NAFLD. Freag et al. designed a triple microchannel chip that maintained stable albumin and urea secretion for more than 2 weeks to visualize the course of pathological changes in NAFL to NASH (Figure 12C).^276^ In addition, they added endothelialized inlets and outlets to the microfluidic device to simulate the flow of drugs through the liver and facilitate collection of metabolites for analysis. Moreover, elafibror, a potential therapeutic drug for NASH, was added into the chip, and it found that elafibror had a certain effect of inhibiting lipid accumulation on the liver‐on‐chip.

#### 5.4.3 Alcoholic liver disease chip

**FIGURE 13:**
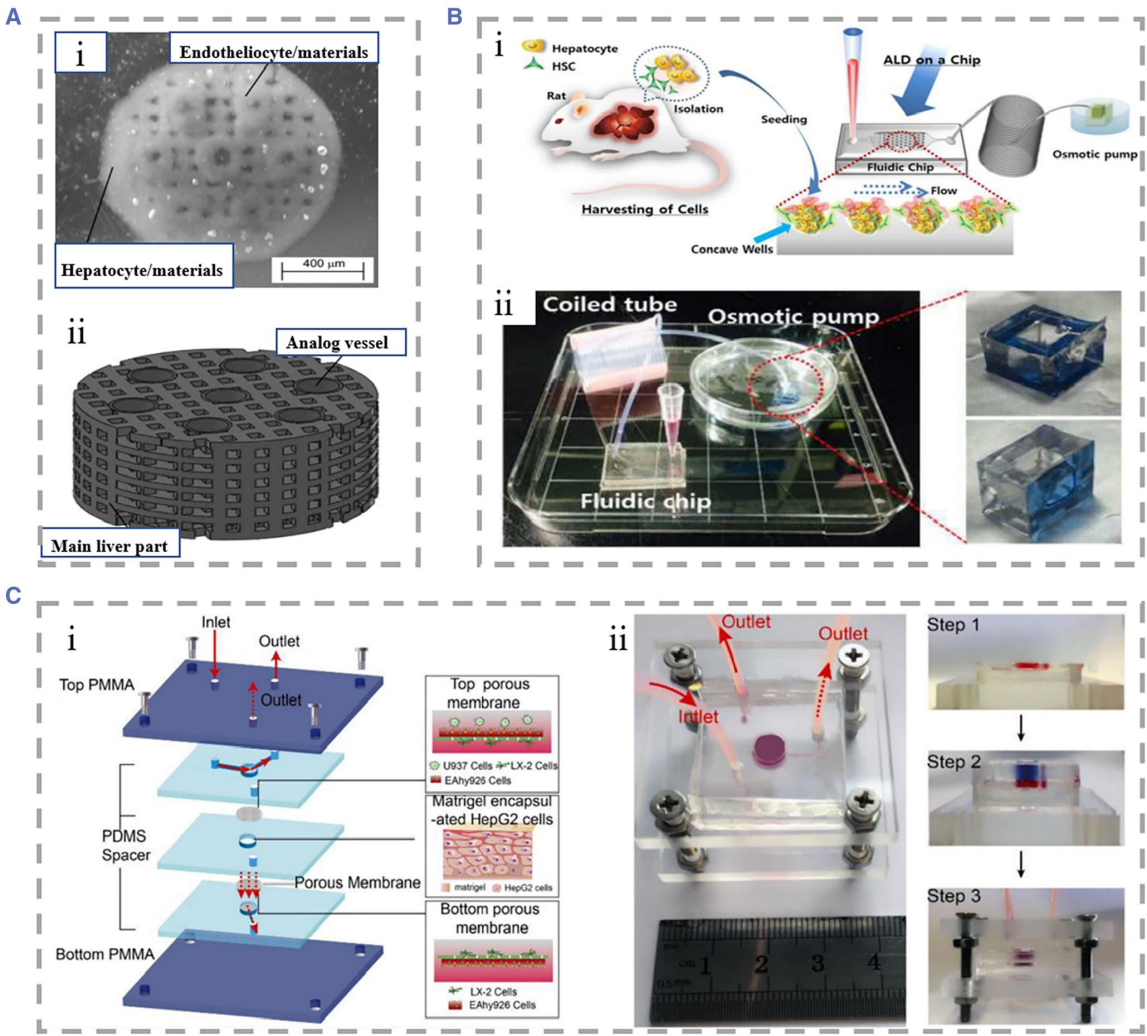
(A) (i) Physical drawing of the liver analog construct. (ii) Design diagram of the liver analog construct. Reproduced under terms of the CC‐BY license.^278^ Copyright 2012, The Authors, published by Springer Nature. (B) (i) Cells were isolated from rats, and they were seeded on a fluidic chip. (ii) Photographs of the whole ALD model. Reproduced with permission.^279^ Copyright 2016, Oxford University Press. (C) (i) ALD models based on four cell line compositions, (ii) Real liver‐on‐a‐chip picture and assembly process. Reproduced with permission.^280^ Copyright 2019, Springer Nature. ALD, alcoholic liver disease.

Alcohol is one of the important causes of liver disease, and ALD has become an important problem faced by both developed and developing countries. The Organ‐on‐chip offers advantages over animal models in terms of lower cost, shorter experimental cycles, and no ethical concerns involved, and thus the organ‐on‐chip offers great advantages in modeling the progression of ALD in vitro. Klassen et al. developed precision‐cut liver slices (PCLS) as a model for ALD.^277^ After 96 h of culture, PCLS eventually developed alcoholic fatty liver. This model contained all cell types of the liver and could well reproduce the process of ethanol‐induced liver injury in vivo. However, this model had a short maintenance time and was not suitable for long‐term observation. Liu et al. constructed an in vitro rat liver analog for ALD studies, in which cultures were exposed to ethanol medium, and the malondialdehyde and nitric oxide concentrations were controlled by the addition of vitamins C and E (Figure 13A).^278^ This system could be used for the study of ALD pathogenesis but was not suitable for mimicking the recovery process of ALD. To establish the full ALD process, including that of alcoholic injury and injury recovery, Lee et al. developed a model of ALD by placing RPHs and HSCs co‐cultured spheroids in a microfluidic platform (Figure 13B).^279^ Then the spheroids were exposed to an ethanol medium for 48 h to mimic the process of liver alcoholic injury. Subsequently, the treated spheroids were cultured on general medium, and fibrous structures produced by HSCs covering the spheroid surface could appear after 3 days, indicating the role of HSCs in the reconstruction of hepatocyte structures in ALD. They observed that the viability of spheroids decreased gradually with increasing alcohol concentration, and the alcoholic liver injury was reversible at alcohol concentrations below 60 μL/mL but irreversible when it was up to 80 μL/mL. Their proposed reversibly and irreversibly injured ALD model had potential for the analysis of ALD pathology and screening of therapeutic drugs. NPCs play an important role in ALD progression, such as LSECs, which secrete inflammatory factors to promote liver injury, and HSCs, which secrete ECM to promote liver fibrosis. However, the cellular behaviors of NPCs have not been fully characterized, and it is of great significance to study their cellular behaviors during the course of ALD. Deng et al. focused on the role of NPCs in ALD.^280^, ^281^ They constructed an ALD model consisting of four cell lines (HepG2, LX‐2, EAhy926, and U937). The chip consisted of two PMMA layers and three PDMS spacer plates separated by two cell seeded polycarbonates. Minipumps set at the inlet of the chip provide the nutrients required for each type of cells. This multicellular structure allowed them to observe the intercellular communication between different types of cells in ALD and helped to investigate the pathophysiology of NPCs in ALD (Figure 13C).

#### 5.4.4 Other liver disease chip

**FIGURE 14:**
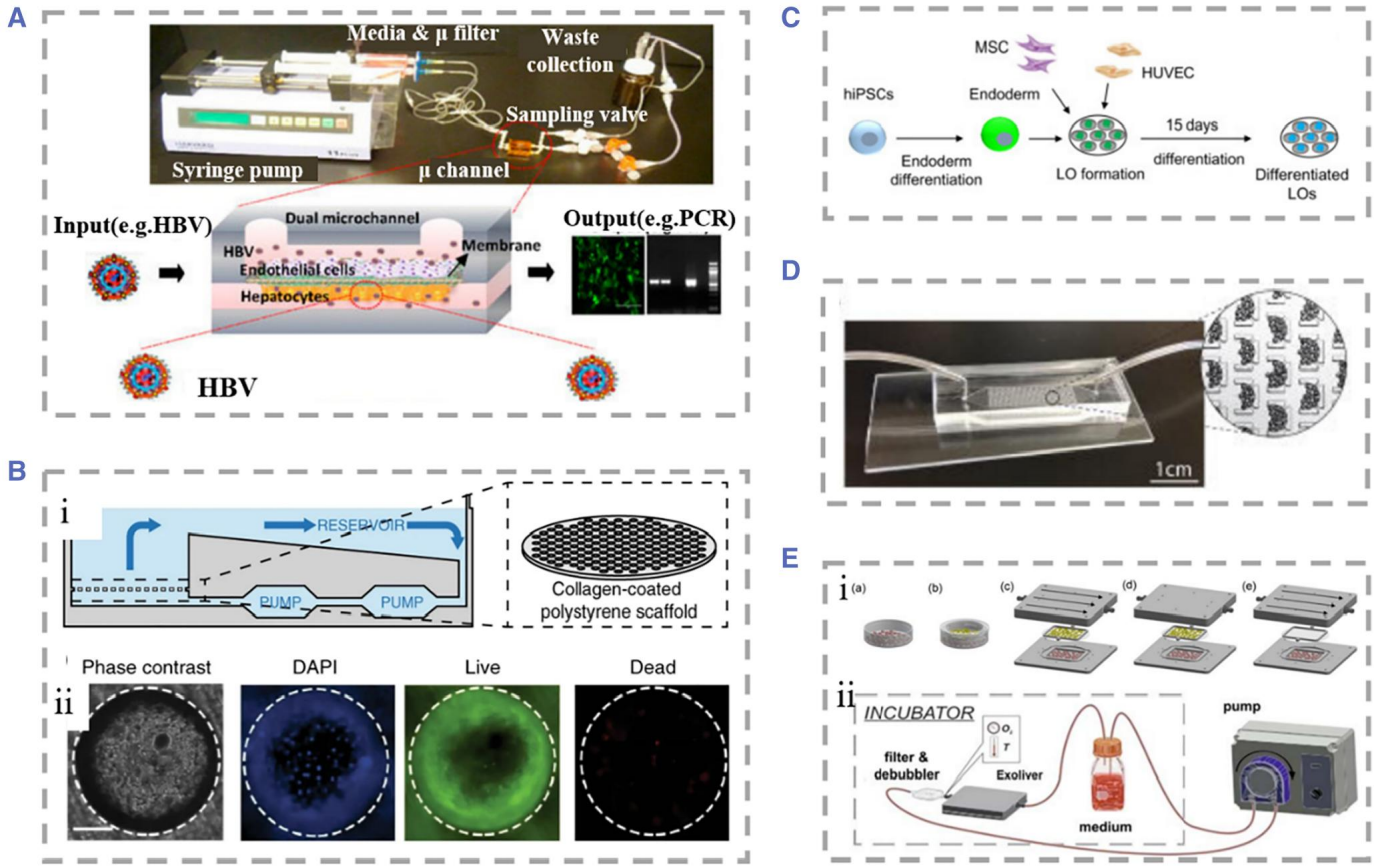
(A) Liver sinusoidal chip with continuous perfusion system and HBV replication circulation. Reproduced with permission.^284^ Copyright 2017, Multidisciplinary Digital Publishing Institute. (B) (i) Schematic representation of the liver‐on‐chip simulating the organization of PHHs on the liver sinusoids. (ii) Cell viability of PHHs seeded on chip. Reproduced under terms of the CC‐BY license.^285^ Copyright 2018, The Authors, published by Springer Nature. (C) Schematic of the process of LOs generated and differentiated from hiPSCs. Reproduced with permission.^255^ Copyright 2018, The Authors, published by Elsevier. (D) Pluripotent stem cell‐based liver organoid chip equipped with peristaltic pumps. Reproduced with permission.^286^ Copyright 2016, The Royal Society of Chemistry. (E) (i) Culture devices for five different experimental conditions. (ii) Schematic representation of the liver sinusoidal chip and circuit components. Reproduced under terms of the CC‐BY license.^287^ Copyright 2018, The Authors, published by John Wiley and Sons. HBV, hepatitis B virus; hiPSCs, human‐induced pluripotent stem cells; LO, liver organoid; PHH, Primary human hepatocytes.

Hepatitis B caused by hepatitis B virus (HBV) is considered a major global health problem. However, the studies on host/pathogen interactions have been limited due to the difficulty in establishing suitable models for the entire viral life cycle and the narrow host range of HBV. With the help of microfluidic technologies, there are many attempts that have been explored to develop more precise in vitro liver models.^282^ Kang et al. first used RPHs and immortalized bovine aortic endothelial cells to establish HBV infection model in 2015.^283^ Since RPHs were not susceptible to direct HBV infection, they used recombinant adenovirus integrated with HBV gene to infect RPHs. In 2017, they proposed a human‐derived hepatocyte and endothelial cell co‐culture model of liver sinusoid based on this model (Figure 14A).^284^ This human‐derived model allowed direct HBV infection and maintained viability for up to 26 days and could be used for studies of chronic HBV infection. In most HBV research models, infection requires high viral titers. Ortega‐Prieto et al. developed a microfluidic primary human hepatocytes culture model for HBV, which was able to study multiplicities of infection 10,000‐fold lower than other models (Figure 14B).^285^ It allowed prolonged culture of PHHs to recapitulate all steps of the HBV life cycle, while also providing a platform for the detection of biomarkers of HBV infection and the validation of the effectiveness of therapeutic interventions. There were also studies developing functional LO chips based on hiPSCs derived endodermal cells, mesenchymal cells, and endothelial cells.^255^ Functional LOs could be maintained for longer periods to support the propagation of HBV and to produce infectious virus. Besides, the hiPSC‐LOs were more susceptible to HBV infection than hiPSC‐HLCs and showed HBV‐induced acute liver failure (Figure 14C).

Schepers et al. proposed an in vitro liver‐on‐chip based on pluripotent stem cells from patients.^286^ They induced iPSCs to generate functional iPSC‐derived HLCs in 3D and maintain function for more than 28 days (Figure 14D). The chip could also be cascaded with other organ chips (e.g., heart, kidney) to form a multi‐organ chip, providing a platform to query patient‐specific liver responses in vitro and test the efficacy of the treatment. Ortega‐Ribera et al. obtained primary hepatocytes and LSECs from the livers of cirrhotic patients and cultured and assessed the hepatocyte viability under five experimental conditions: monoculture, co‐culture with LSECs, co‐culture with LSECs with uniform shear stress (optimal condition), co‐culture without shear stress, and monoculture with hepatocytes with indirect flow stimulation (Figure 14E).^287^ This device mimicked the in vivo liver sinusoidal microenvironment, providing a new approach for the development of targeted drugs and personalized therapy for patients.

## 6 CONCLUSION AND OUTLOOK

Biomimetic microfluidic organ‐on‐a‐chip is an emerging technology based on tissue engineering, emerging materials, and microfluidic technology with unique advantages in several fields. A number of liver‐on‐a‐chip models have been developed that form the basis of this review. Our aim is to give the reader an overview of liver physiology and the current status and significance of liver‐on‐a‐chip studies. In this review, we first briefly describe the structure, cellular composition, and physiological functions of the liver. Then we review the current state of liver‐on‐chips from three aspects: culture environment of liver‐on‐chips, cell system, in vitro liver models. Finally, we discuss examples of liver‐on‐chips applications in several aspects.

Although liver‐on‐chip research has made remarkable progress, it currently still faces many challenges. To solve this problem, the following points are worth noting. Firstly, although various kinds of liver‐on‐chips have been developed, they are still far to the real liver. At present, liver‐on‐chips can only simulate partial structure or limited functions of liver. Even though the metabolites of many drugs need to be excreted by bile acids, most of the chips lack the bile canalicular structure and the pathway of bile acid excretion, which is disadvantageous to the study of drug metabolism and the maintenance of long‐term liver culture, which requires researchers to consider completely and systematically when designing a liver chip in the future. Moreover, due to the limitations of machining conditions and the number of cell stacking layers, the liver‐on‐chips cannot fully mimic the fine structure of the liver in vivo. In addition, as an ideal candidate for cell culture, PDMS is nontoxic and has high gas permeability. But evaporation of moisture may lead to changes in the osmotic pressure of PDMS‐based medium, and the high gas‐permeability also makes the oxygen tension of the target area difficult to control. With the advent of new chip models, the invention of new ideal media materials and the combination of compartments that mimic liver specific functions, this issue is also likely to be solved in the future. Secondly, there are limited sources of liver cells suitable for constructing liver‐on‐chips. The commonly used cells are immortalized hepatocytes, primary hepatocytes, etc., which have their own disadvantages and limited access to fabricate the chips. Recently, research on stem cells has made good progress, indicating that mutant human embryonic stem cells and iPSC‐Heps cells are a possible alternative solution. Thirdly, efficient methods for the production of liver‐on‐chips with large‐scale are lacking. The advantage of the liver‐on‐chip is to utilize a small number of cells to achieve mimicry of liver ultrastructure at the microscale. Nevertheless, patients waiting for liver transplantation require a large amount of liver tissue, while there are still limited ways to rapidly scale up liver‐on‐chips to the required volume. In the future, multiple biosensor integration will make up for the deficiencies of liver chips in readout and throughput, and improvements in the fabrication process and the use of high‐tech detection technology will also benefit commercial liver chips in reducing the cost and increasing the yield.

Liver‐on‐chips hold great value as a novel platform for preclinical drug development, the study of mechanisms of liver disease and so on, with the potential to replace animal models in food or drug development in the future. Despite many problems awaiting resolution, it is believed that the liver‐on‐chip technology will mature more and more with further development in the fields of biomimicry, materials, and machining, the above issues are promising to be explored, and the research and development of liver‐on‐chips will have a bright future and new era.

## AUTHOR CONTRIBUTIONS

Huan Wang conceived the conceptualization and designed the paper. Linjie Qiu and Huan Wang wrote and revised the paper. Bin Kong and Tiantian Kong participated in discussion and revised the paper.

## CONFLICT OF INTEREST STATEMENT

The author declares that there is no conflict of interest that could be perceived as prejudicing the impartiality of the research reported.
